# A P2RY12 deficiency results in sex-specific cellular perturbations and sexually dimorphic behavioral anomalies

**DOI:** 10.1186/s12974-024-03079-7

**Published:** 2024-04-15

**Authors:** Ogochukwu J. Uweru, Akhabue K. Okojie, Aparna Trivedi, Jordan Benderoth, Lauren S. Thomas, Georgia Davidson, Kendall Cox, Ukpong B. Eyo

**Affiliations:** 1 Center for Brain Immunology and Glia, University of Virginia, Charlottesville, VA USA; 2 Department of Neuroscience, University of Virginia, Charlottesville, VA USA; 3 Department of Biomedical Engineering, University of Virginia, Charlottesville, VA USA; 4 North Carolina Agricultural and Technical State University, Greensboro, NC USA; 5 Neuroscience Graduate Program, University of Virginia, Charlottesville, VA USA

**Keywords:** Microglia, P2RY12, Sex differences, Locomotion, Behavior

## Abstract

Microglia are sexually dimorphic, yet, this critical aspect is often overlooked in neuroscientific studies. Decades of research have revealed the dynamic nature of microglial-neuronal interactions, but seldom consider how this dynamism varies with microglial sex differences, leaving a significant gap in our knowledge. This study focuses on P2RY12, a highly expressed microglial signature gene that mediates microglial-neuronal interactions, we show that adult females have a significantly higher expression of the receptor than adult male microglia. We further demonstrate that a genetic deletion of P2RY12 induces sex-specific cellular perturbations with microglia and neurons in females more significantly affected. Correspondingly, female mice lacking P2RY12 exhibit unique behavioral anomalies not observed in male counterparts. These findings underscore the critical, sex-specific roles of P2RY12 in microglial-neuronal interactions, offering new insights into basal interactions and potential implications for CNS disease mechanisms.

**Graphical Abstract:**

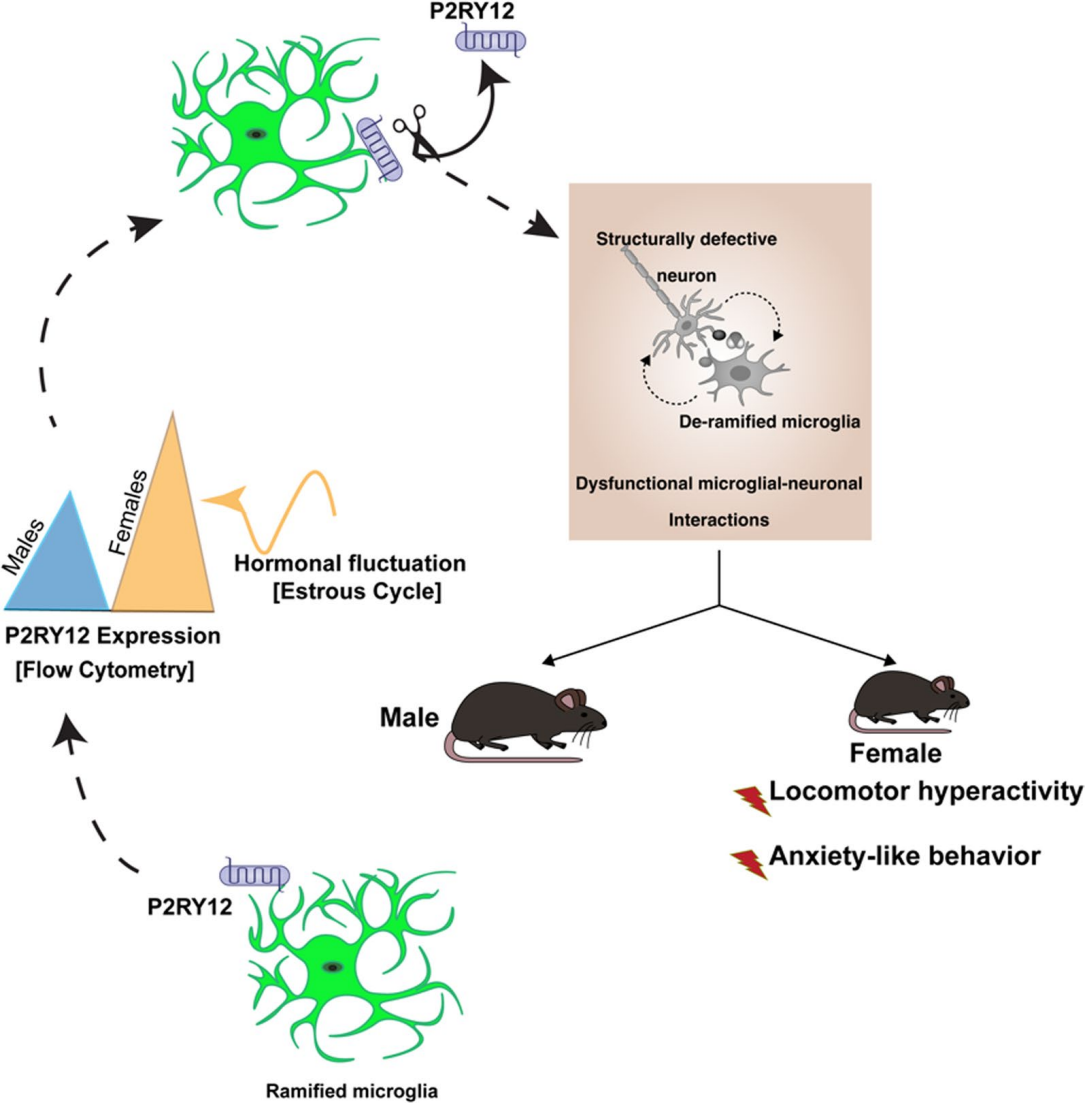

**Supplementary Information:**

The online version contains supplementary material available at 10.1186/s12974-024-03079-7.

## Highlights

- Microglial P2RY12 shows a greater protein expression in adult females than adult males
- Microglial P2RY12 deficiency triggers morphological perturbations, especially in females
- Microglial P2RY12 deficiency triggers sex-specific dendritic spine alterations
- Microglial P2RY12 deficiency triggers sexually dimorphic behavioral anomalies

**Supplementary Information:**

The online version contains supplementary material available at 10.1186/s12974-024-03079-7.

## Background

In mammals, males and females exhibit some salient differences in their biological makeup [2, 36]. These distinctions, manifesting at the molecular, cellular, and behavioral levels, may underpin why each sex experiences a disparate susceptibility to neurodegenerative, infectious, and neuropsychiatric diseases [27, 54]. One pivotal cellular player in this arena, is the microglial cell.

Microglia, the primary resident immune cells of the brain, have been spotlighted as vital agents in both health and disease contexts of the central nervous system (CNS) [15, 25, 33, 34, 42]. Their role in neuroinflammation is emerging as crucial in the trajectory of many CNS disorders [18, 22, 39, 48, 60]. Moreover, microglia are not just bystanders; they actively shape the brain’s development and are intrinsic to maintaining tissue homeostasis [40, 50, 52]

An integral part of how microglia help to maintain tissue homeostasis and physiology as to do with their interactions with neurons [53, 59]. While their mechanisms of action and interaction with neurons remain areas of active research, there is a growing intrigue around the molecules that mediate this microglia-neuron crosstalk or communication. One of such molecules that is rapidly gaining attention in the field is P2RY12—a microglial signature protein that is encoded by the *P2ry12* gene.

The P2RY12 is evolutionarily conserved, highly, and selectively expressed microglial receptor involved in their interactions with the CNS extracellular environment [21, 24]. Studies have illuminated its function in directing microglial processes and in providing protective roles in contexts of injury [12, 19, 23]. Pertinently, recent studies [12–14] demonstrate that P2RY12 not only facilitates interactions between microglial processes and neuronal elements but is also important for the development and maintenance of specialized somatic purinergic interaction sites between microglia and neuronal somas, both during development and in adulthood. This indicates that P2RY12 likely plays a sustained role in orchestrating microglia-neuron communication throughout life.

A growing observation in the field is the sexually dimorphic nature of microglia [7, 29, 30, 58]. Their distinct roles and functions in males versus females might underpin certain behavioral and anatomical differences [44, 55, 57]. While P2RY12’s protective effects are increasingly being recognized in some CNS injuries, a significant gap exists in understanding P2RY12’s function within a sex-specific context.

In this study, we aim to address this gap by examining the consequences of a P2RY12 deficiency on histological and behavioral outcomes. Our research specifically targets the significance of P2RY12 in determining microglial phenotypes across sexes. As a foundational step, we analyzed the expression of P2RY12 protein in microglia across both male and female mice from early postanal development through to adulthood. We report that the expression of the receptor differs between females and males, a finding observed only in adulthood. This prompted us to examine both histological and behavioral aspects of P2RY12 deficiency in adult mice of both sexes.

Our investigations revealed effects of the P2RY12 deficiency that do not depend on the sex i.e. microglial density. However, we discovered that the P2RY12 deficiency impacts microglial morphology and neuronal spine density in a sex-specific manner and these structural changes were associated with distinct behavioral patterns. Female mice exhibited unique changes in locomotion and anxiety-like behaviors, suggesting that some aspects of P2RY12 function in microglial-neuronal interactions are sex-specific and critical for understanding brain function and behavior. These findings underscore the importance of sex as a critical factor in neuro-immune interactions, revealing previously underappreciated, sex-specific dimensions of P2RY12’s role in the brain.

### Sex-dependent expression of P2RY12 in microglia

**Fig. 1:**
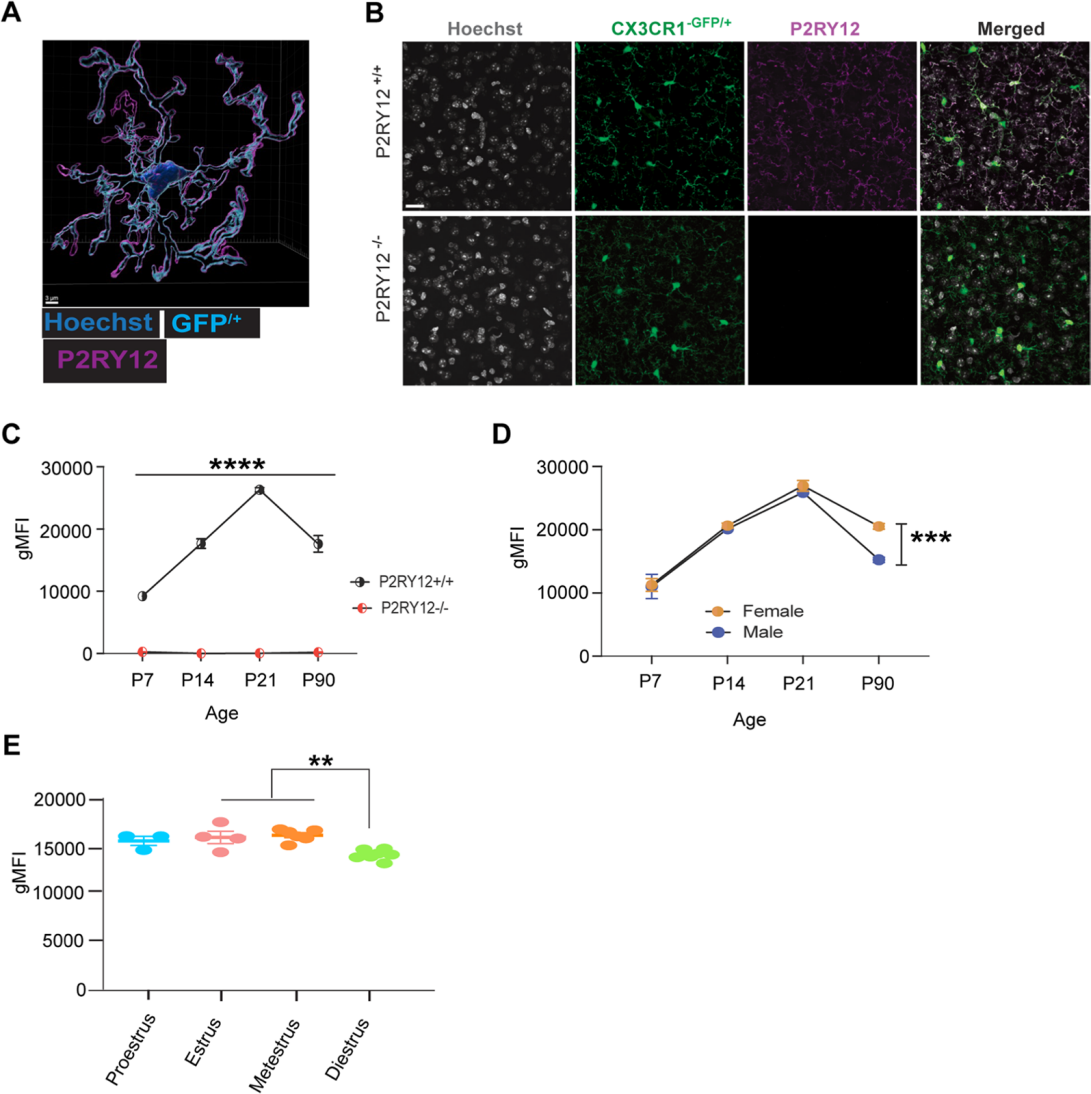
Sex-dependent expression of P2RY12 in adult microglia. **A** Shows a three-dimensional reconstructed image of microglia demonstrating the specificity of P2RY12 antibody labeling in CX3CR1^−GFP/+^ reporter mice scale bar: 3 µm. **B** Provides sample cortical immunofluorescent images confirming the absence of P2RY12 reactivity in adult CX3CR1^−GFP/+^: P2RY12^−/−^ mice scale bar: 20 µm. (**C**) Presents flow cytometric analysis verifying the absence of P2RY12 from development through to adulthood in CX3CR1^−GFP/+^: P2RY12^−/−^ mice. **D** Details P2RY12 expression in microglia by sex, and (**E**) document P2RY12 expression in female mice across the estrous cycle. Data in panel C and D were analysed using student’s t-test, and data in **E** were analysed using one-way ANOVA with Tukey’s post hoc test. Data presented are reported as mean ± SEM. A *p*-value < 0.05 denotes statistical significance. N = 3–5 for **C** N = 4–9 per group for **D**, and N = 3–7 per group for panel E. **p signifies* p* < 0.01; ***p signifies* p* < 0.001, ****p signifies* p* < 0.0001, with the symbols indicating levels of statistical significance

For the assessment of P2RY12 expression pattern we used transgenic mice expressing GFP driven by the endogenous *Cx3cr1* locus (CX3CR1^GFP/+^ or P2RY12^−/−^:CX3CR1^GFP/+^). Immunohistochemistry (IHC) P2RY12 shows colocalized expression with GFP^+^ cells in wildtype mice and lack of expression in P2RY12^−/−^ adult mice (Fig. 1A, B). Examination of cortical P2RY12 expression by immunohistochemistry failed to show any distinctions in wildtype male compared to female mice (Additional file 1: Figure S1C, D). But since P2RY12 is highly expressed in microglia and IHC may not be able to detect subtle differences of expression between the sexes, we employed flow cytometry that has more sensitive detection capabilities.

Given that microglial sexual dimorphism has developmental origins [7, 31] and there is little knowledge about P2RY12 receptor expression pattern from development to adulthood, we examined the trajectory of P2RY12 expression from development into adulthood. We began our analysis at P7. At this stage, female microglia are irrevocably committed to the process of feminization and less sensitive to exogenous testosterone; concurrently, testosterone levels in males have significantly decreased [37]. We continued our analysis through to P90 when the animals are fully mature.

Using flow cytometry on whole brain, we confirmed that P2RY12^−/−^ mice lack expression of P2RY12 across all ages, from development to adulthood (Fig. 1C). Interestingly, we observed an upward trend in the expression level of P2RY12 from postnatal day 7 (P7), peaking at postnatal day 21 (P21), before declining to lower levels in adults (Fig. 1D). In adulthood, we discovered a significant difference (*p* < 0.001) in P2RY12 protein levels between males and females, with females showing higher P2RY12 protein levels than males (Fig. 1D). This sex-specific disparity in P2RY12 expression aligns with the distinct male and female microglial transcriptomic profiles available at Microglia-Seq database. Interestingly, within the female population, P2RY12 expression not only varied across the estrus cycle in females but also peaks specifically during the estrus and metestrus phases (Fig. 1E). This pattern suggests that hormonal fluctuations significantly influence P2RY12 levels, adding a layer of complexity to its regulation. These findings collectively underscore the sex-dependent regulation of P2RY12 in adulthood, highlighting the nuanced interplay between hormonal cycles and microglial functions, setting the foundation for a deeper understanding of microglial-neuronal interactions in the brain. Building on this groundwork, our subsequent investigations will delve into the consequences of the genetic deletions of P2RY12, exploring its histological and behavioral outcomes.

### P2RY12-deficiency results in an increase in microglial population density

**Fig. 2:**
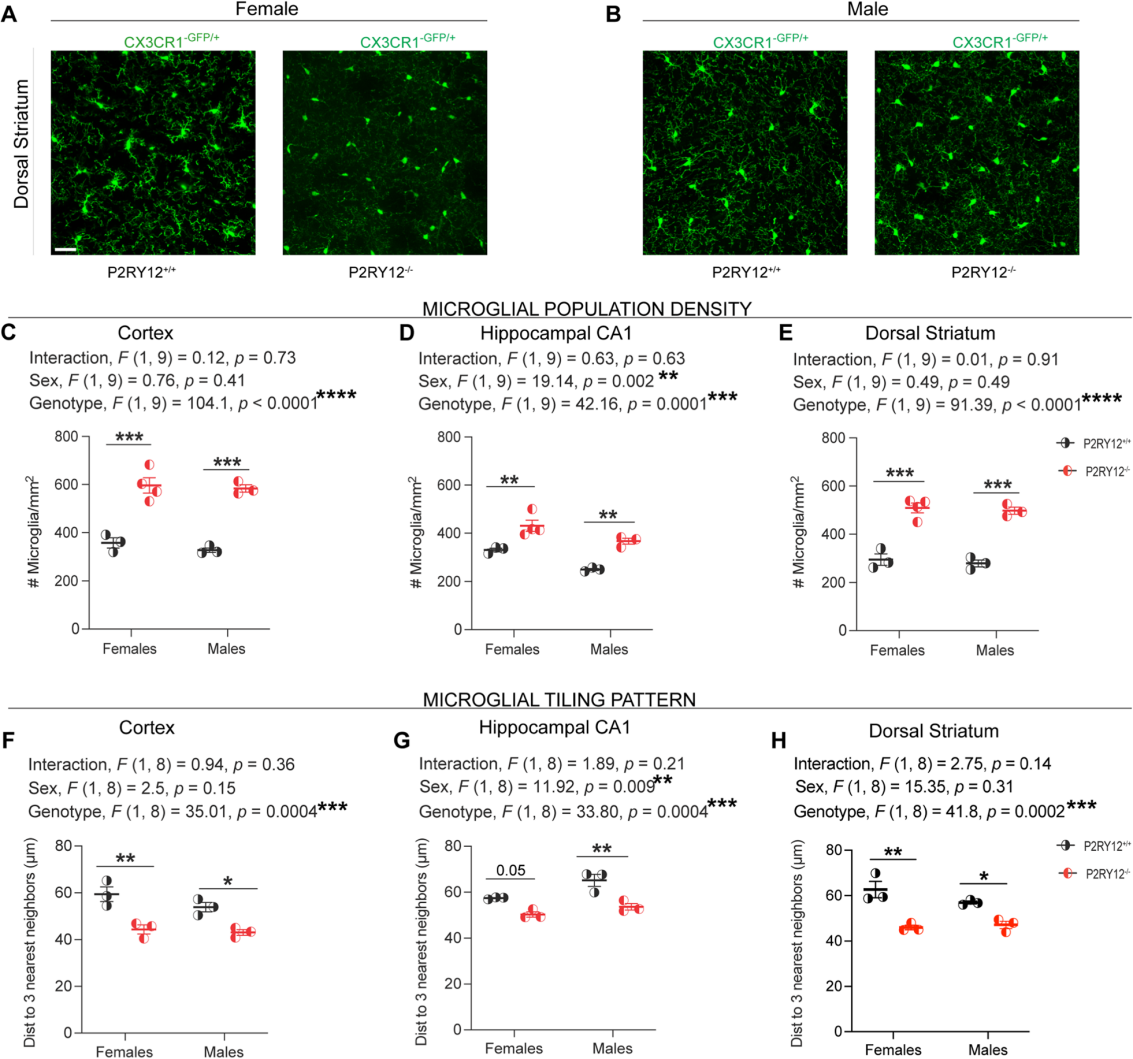
P2RY12 deficiency results in an increase in microglia population density. **A**, **B** Display representative maximum intensity projections showcasing microglia density in the dorsal striatum of adult female and male mice. Scale bar: 20μm.  **C**–**E** Indicate an increase in microglia density in the cortex, hippocampal CA1, and dorsal striatum for both female and male mice lacking P2RY12. **F**–**H** Demonstrate that the average distance to the three nearest neighbors is reduced in the specified brain region in both female and male P2RY12-deficient mice. Data analysis was performed with 2-way ANOVA to assess the effects of both genotype and sex and presented as mean ± SEM. Tukey’s multiple comparisons test was applied for post hoc analysis. A *p*-value < 0.05 was considered significant, with levels of significance indicated by: *p < 0.05*,* **p < 0.01, ***p < 0.001, ****p < 0.0001. N = 3–4 mice per group

Understanding alterations in microglial density is important for neurobiological processes, particularly in relation to P2RY12’s role in microglial-neuronal communication and its implication for neurodegenerative diseases and neuropsychiatric disorders. Considering that recent work has confirmed the presence of P2RY12-mediated microglial-neuronal crosstalk in the cortex, hippocampus, and striatum [12], our analysis focused on these three regions. Using the transgenic mice expressing GFP driven by the endogenous *Cx3cr1* locus i.e., CX3CR1^GFP/+^ or P2RY12^−/−^:CX3CR1^GFP/+^ mice (Fig. 2A, B, our data show an increase in microglial density in the cortex (genotype, *F*_(1, 9)_ = 104.1, *p* < 0.0001, Fig. 2C), hippocampal CA1 (genotype, *F*_(1, 9)_ = 42.16, p = 0.0001, Fig. 2D), and dorsal striatum (genotype, *F*_(1, 9)_ = 91.39, *p* < 0.0001, Fig. 2E) in the P2RY12-deficient mice when compared to their P2RY12-sufficient littermates. No significant sex-genotype interactions (cortex: interaction, *F*_(1, 9)_ = 0.12, *p* = 0.73, Fig. 2C; hippocampal CA1: interaction, *F*_(1, 9)_ = 0.63, *p* = 0.63, Fig. 2D; dorsal striatum: interaction, *F*_(1, 9)_ = 0.01,* p* = 0.91, Fig. 2E) were observed. Interestingly, while a main effect of sex was observed in the hippocampal CA1 (sex, *F*_(1, 9)_ = 19.14, *p* = 0.002, Fig. 2D), it was absent in the cortex (sex, *F*_(1, 9)_ = 0.76, *p* = 0.41, Fig. 2C) and dorsal striatum (sex,* F*_(1, 9)_ = 0.49, *p* = 0.49, Fig. 2E), suggesting region-specific differences in microglial response to a P2RY12 deficiency. Additionally, our post hoc analysis shows significant increases in microglia density in both male and female P2RY12-deficient mice in the cortex (females [*p* = 0.0002], males [*p* = 0.0002]), hippocampal CA1 (females [*p* = 0.008], males [*p* = 0.005]) and the dorsal striatum (females [*p* = 0.001], males [*p* = 0.0001]) when compared to their P2RY12-sufficient littermates.

Microglia somata are largely stationary with uniform three-dimensional distribution that reflects a tiling pattern with cell-to-cell distances of ~ 50–60 μm (in the cortex) [41]. Given the significant increase in microglial density we observed in P2RY12-deficient mice, it stands to reason that their territorial organization, reflected by their tiling pattern, might also be altered. By analyzing the average distance to the nearest three neighbors—a method that provides a robust measure of spatial distribution—we observed notable changes in the tiling pattern of microglia across various brain regions. Specifically, there was a significant genotype effect on the spatial distribution in the cortex (genotype,* F*_(1, 8)_ = 35.01, *p* = 0.0004, Fig. 2F), hippocampal CA1 (genotype, *F*_(1, 8)_ = 33.80, *p* = 0.0004, Fig. 2G), and the dorsal striatum (genotype, *F*_(1, 8)_ = 41.8, *p* = 0.0002, Fig. 2H). Additionally, a significant sex effect was observed in the hippocampal CA1 (sex, *F*_(1, 8)_ = 11.92, *p* = 0.009, Fig. 2G), but not in the cortex (sex, *F*_(1, 8)_ = 2.5, *p* = 0.15, Fig. 2F) or dorsal striatum (sex, *F*_(1, 8)_ = 1.19, *p* = 0.3, Fig. 2H). No significant sex-genotype interactions were detected in the cortex (interaction, *F*_(1, 8)_ = 0.94, *p* = 0.36, Fig. 2F), the hippocampal CA1 (interaction, *F*_(1, 8)_ = 1.89, *p* = 0.63, Fig. 2G) or dorsal striatum (interaction, *F*_(1, 8)_ = 2.75, *p* = 0.41, Fig. 2H). Furthermore, our post hoc pair-wise comparisons show that both male and female P2RY12-deficient microglia in the cortex (females [*p* = 0.005]; males [*p* = 0.03], Fig. 2H), hippocampal CA1 (females [*p* = 0.05]; males [*p* = 0.004], Fig. 2G), and dorsal striatum (females [*p* = 0.002]; males [*p* = 0.04], Fig. 2H) exhibit a significant decrease in the average distance to their nearest three neighbors compared to their P2RY12-sufficient counterparts.

Collectively, our findings reveal that the absence of P2RY12 disrupts the normal density and tissue tiling patterns of microglia in both males and females, underscoring a critical role of P2RY12 in preserving microglial homeostatic population density and their three-dimensional territorial organization.

### P2RY12-deficient female microglia show pronounced de-ramification

**Fig. 3:**
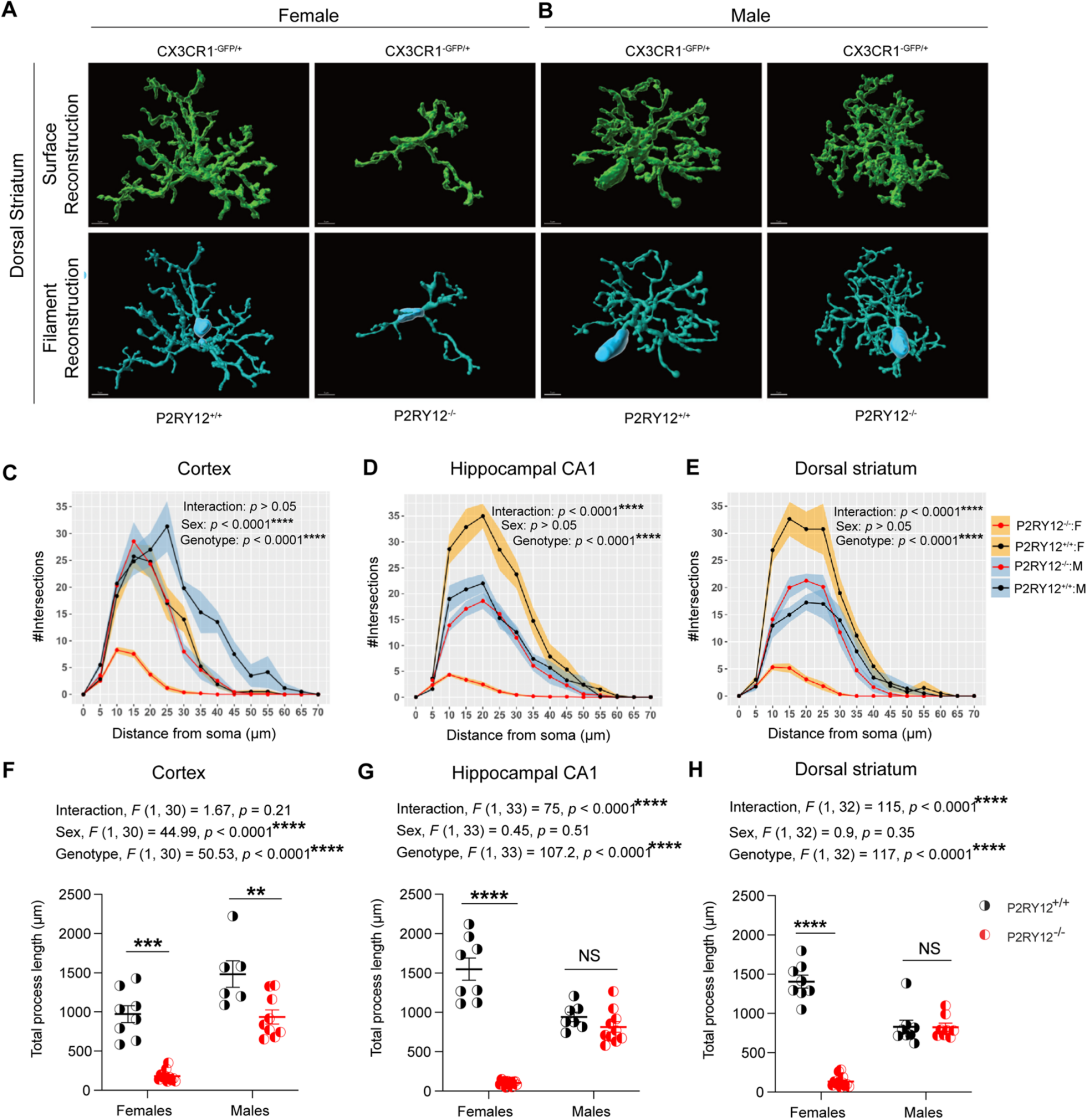
P2RY12-deflicient female microglia show pronounced de-ramification. **A**, **B** Representative three-dimensional reconstructions (surface and filament) of adult male and female microglia using Imaris are shown, highlighting detailed structural differences. Scale bar: 5 µm. **C**–**E** Utilizes Sholl analysis to compare microglia complexity in the cortex (frontal cortex-layer 5), hippocampal CA1, and dorsal striatum illustrating sex and regional variations in microglial morphology. **F**–**H** Demonstrate significant reductions in total microglia process length per cell in P2RY12-deflicent microglia across difference brain regions and sex, with emphasis on varied impact in females and males. Data were analysed by using 2-way ANOVA, with results expressed as mean ± SEM. Shading in panel **C-E** represent SEM.Tukey's multiple comparisons test was utilized for post hoc analysis. Data points are 6–12 intact microglia per brain region, derived from 2 to 3 mice per group. Significance levels: *p denotes *p* < 0.05, **p denotes *p* < 0.01 ***p denotes *p* < 0.001 ****p denotes *p* < 0.0001

Given that microglia are morphologically plastic [16, 41] and sexually dimorphic in the complexity of their process arborization [11], we investigated whether P2RY12 deficiency affects microglial process length and complexity in a sex-specific manner. Through high (40x) magnification confocal microscopy imaging and 3D Sholl analysis using Imaris on CX3CR1^GFP/+^ and P2RY12^−/−^:CX3CR1^GFP/+^ mice, we discovered that P2RY12-deficient female microglia exhibit significant de-ramification, characterized by reduced process branching, in the cortex, hippocampal CA1, and dorsal striatum. In contrast, P2RY12-deficient males showed similar de-ramification only in the cortex, as compared to their respective P2RY12-sufficient counterparts (Fig. 3A–E). Through 3D reconstruction of individual cells, we discovered that P2RY12-deficiency leads to a significant reduction in total process length, notably in a sex-specific manner. This effect was pronounced in the hippocampal CA1 (interaction, *F*_(1, 33)_ = 75, *p* < 0.0001, Fig. 3G) and the dorsal striatum (interaction,* F*_(1, 32)_ = 115, *p* < 0.0001, Fig. 3H) of females, but was not observed in the cortex (interaction, *F*_(1, 30)_ = 1.67, *p* = 0.21, Fig. 3F). Additionally, significant genotype effects were observed across all examined regions, indicating a general impact of P2RY12 deficiency on microglial morphology: cortex (genotype, *F*_(1, 30)_ = 50.53,* p* < 0.0001, Fig. 3F), hippocampal CA1 (genotype, *F*_(1, 33)_ = 107.2, *p* < 0.0001, Fig. 3G), and dorsal striatum (genotype,* F*_(1,32)_ = 117, *p* < 0.0001, Fig. 3H). Interestingly, a significant main effect of sex was found in the cortex (sex, *F*_(1, 30)_ = 44.99, *p* < 0.0001, Fig. 3F), but not in the hippocampal CA1 (sex, *F*_(1, 33)_ = 0.45, *p* = 0.51, *p* = 0.51, Fig. 3G) or dorsal striatum (sex, *F*_(1, 32)_ = 0.9, *p* = 0.35, Fig. 3H). Our post hoc pair-wise comparisons further revealed a dramatic reduction in total process length among P2RY12-deficient female microglia across the cortex (*p* = 0.0001, Fig. 3F), hippocampal CA1 (*p* < 0.0001, Fig. 3G), and dorsal striatum (*p* < 0.0001, Fig. 3H), with males showing a similar reduction only in the cortex (*p* = 0.003, Fig. 3F). These results suggest that P2RY12 regulates microglial morphology in a sex-specific manner, as indicated by the dramatic reduction in the complexity of P2RY12-deficient female microglia. These findings underscore the critical role of P2RY12 in modulating microglial morphology and highlight a marked sex-specific vulnerability in females, suggesting that the absence of P2RY12 leads to considerable changes in microglial structural complexity.

### Sex-specific spine density alterations in P2RY12-deficient mice

Recent work from Cserép et al. [12] has unveiled dynamic P2RY12-mediated interactions between microglial processes and different parts of neurons across the cortex, hippocampus, and striatum. This suggests a pivotal role of microglia in modulating neuronal structure in both superficial and deep regions of the brain. Given the critical involvement of P2RY12 in microglial-neuronal interactions, we aimed to investigate the extent to which P2RY12-induced microglial perturbations impact neuronal population and spine densities across sex. Understanding these interactions across sex is crucial, as alterations in neuronal architecture can significantly affect neural circuit, potentially influencing behavior and susceptibility to CNS disorders.

**Fig. 4:**
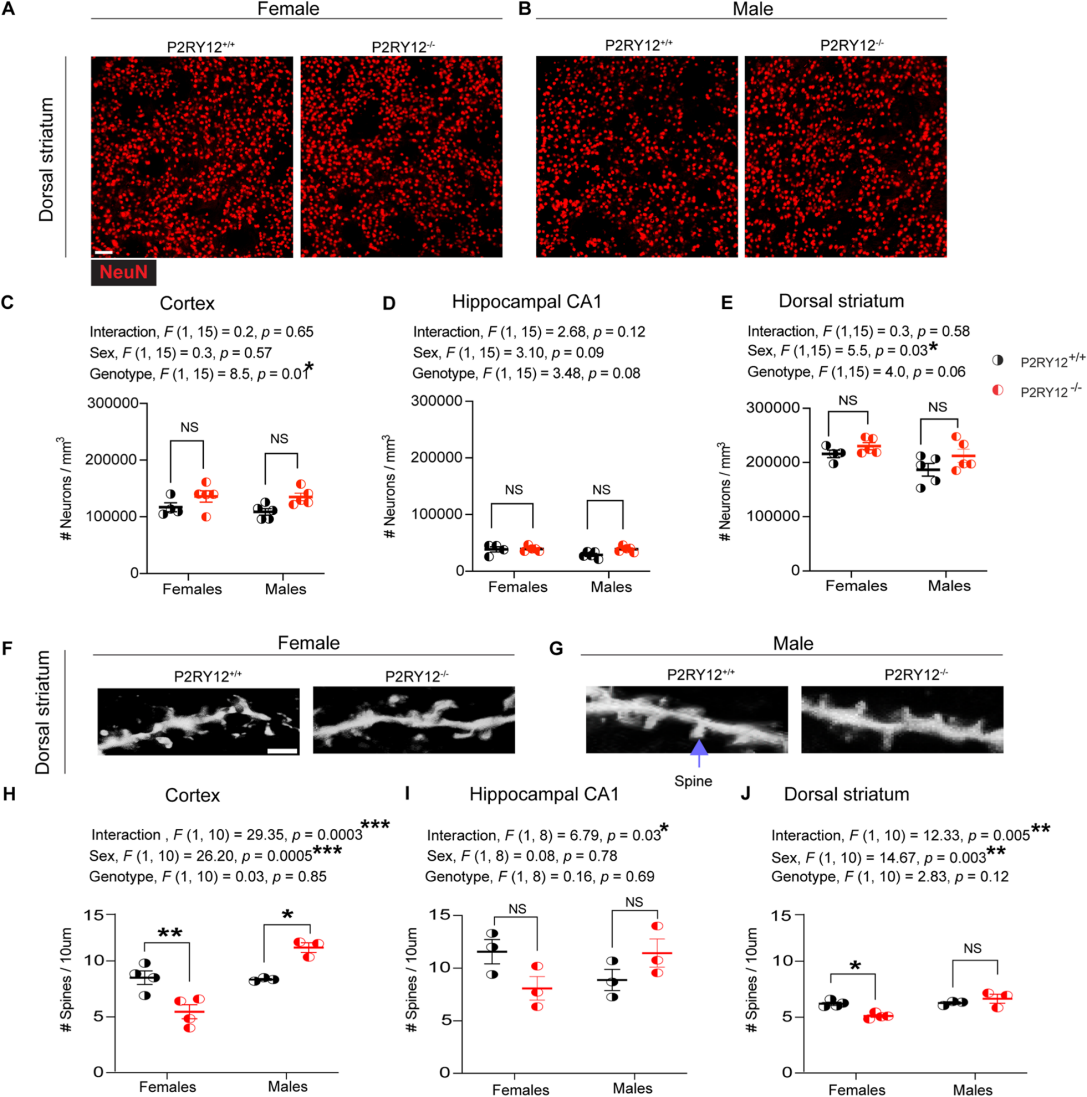
Sex-specific alteration in dendritic spine density. **A**, **B** Representative NeuN-stained images of neuronal density in the dorsal striatum of both sexes. Scale bar: 50 µm. **C** Genotype significantly affects cortical neuronal density. **D** Neuronal density in the hippocampus CA1 region consistent across groups. **E** Dorsal striatum neuronal density varies by sex. **F**, **G** Glogi-stained images of secondary order dendritic segment with spines in the dorsal striatum, highlighted by purple arrow pointing to dendritic spines, scale bar: 5 µm. **H** Cortical spine density indicate significant difference by sex and sex-genotype interaction, with decreases in P2RY12-deficient female and increases in deficient males compared to their sufficient counterparts. **I** Hippocampal CA1 spine density reveals a significant sex-genotype interaction. **J** Dorsal striatum spine density shows significant sex and sex-genotype interaction, with a decrease in P2RY12-deficient female but no change in deficient males compared to sufficient counterparts. Statistical analysis was conducted using 2-WAY ANOVA, with Tukey’s post hoc test for specific comparisons. Significance was set as *p* < 0.05. Data are presented as mean ± SEM for N = 3–5 mice per group. Significance levels: *p denotes p < 0.05, **p < 0.01, ***p < 0.001

In our investigation into neuronal population density through NeuN immunohistochemistry (Fig. 4A, B), we identified significant genotype-dependent differences in the cortex (genotype, *F*_(1,15)_ = 8.57, *p* = 0.01, Fig. 4C), suggesting genetic differences significantly influence neuronal population density. While exploring the hippocampal CA1 region, we observed trends towards significance for both sex (sex, *F*_(1, 15)_ = 3.10, *p* = 0.09, Fig. 4D) and genotype (genotype, *F*_(1, 15)_ = 3.48, *p* = 0.08, Fig. 4D), indicating potential biological effects on neuronal density. Notably, the dorsal striatum exhibited a significant sex difference in neuronal density (sex, *F*_(1,15)_ = 5.5, *p* = 0.03, Fig. 4E) and a nearly significant genotype effect (genotype, *F*_(1, 15)_ = 4.0, *p* = 0.06 Fig. 4E), highlighting a critical role of sex and genotype in neuronal population density in this region of the brain.

Using the Golgi staining technique for spine density analysis (Fig. 4F, G), our data reveal significant sex-genotype interactions in the cortex (interaction, *F*_(1, 10)_ = 29.35, *p* = 0.0003, Fig. 4H), hippocampal CA1 (interaction, *F*_(1, 8)_ = 6.79, *p* = 0.03, Fig. 4I), and the dorsal striatum (interaction, *F*_(1, 10)_ = 12.33, *p* = 0.005, Fig. 4J). Interestingly, we also find a main effect due to sex in the dorsal striatum (sex, *F*_(1, 10)_ = 14.67, *p* = 0.003, Fig. 4J) and the cortex (sex, *F*_(1, 10)_ = 26.20, *p* = 0.0005, Fig. 4H). Our pair-wise comparisons show that P2RY12-deficient females have a significant reduction in their dendritic spine density in the cortex (*p* = 0.007, Fig. 4H) and the dorsal striatum (*p* = 0.01, Fig. 4J). While the dendritic spine density in the P2RY12-defcient male dorsal striatum remains unaffected, we find that they have an increase in their cortical spine density (*p* = 0.03, Fig. 4H). Collectively, our data show sex-specific alterations in spine density in the dorsal striatum and cortex, but not in neuronal density, thus suggesting that the observed microglial perturbations due to the loss of P2RY12 may have a neuroanatomical impact.

### P2RY12-deficient females display locomotor hyperactivity

To evaluate the behavioral impact of sex-specific cellular alterations observed in P2RY12-deficient mice, we conducted a set of behavioral tests, including the open field locomotor test, the elevated plus maze test, and the accelerated rotarod test. The focus on these assays is especially pertinent due to the sex-specific cellular changes noted in the hippocampus and dorsal striatum— regions critical for anxiety-like behaviors and locomotor control, respectively [28, 61]. This approach aims to determine if the cellular disruptions translate into discernible behavioral phenotypes, thereby shedding light on the functional consequences of P2RY12 deficiency.

**Fig. 5:**
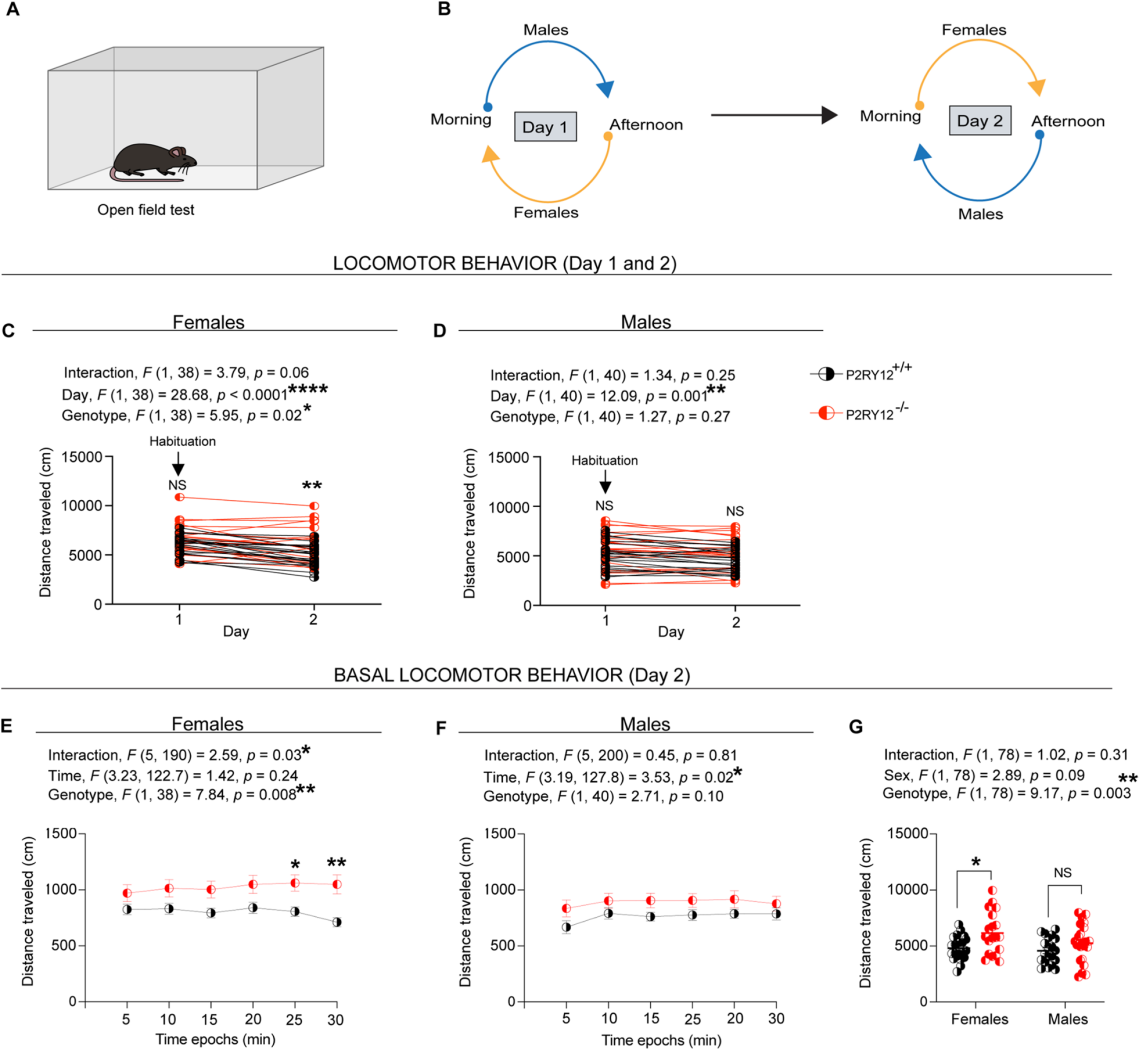
P2RY12-deficient females display locomotor hyperactivity. **A** A sketch of the open field arena. **B** Schematic of the open field experimental design, conducted twice daily for two consecutive days. **C** P2RY12-deficient females exhibit increased cumulative distance and lack inter-session habituation. **D** No difference in cumulative distance traveled by P2RY12-deficient males compared to their P2RY12-sufficient littermates. **E** P2RY12-deficient females show increases basal locomotor on day 2 and impaired intra-session habituation. **F** Basal locomotor activity in P2RY12-deficient males is compared to P2RY12-sufficient counterparts. **G** Genotype significantly affects basal locomotion, with P2RY12-deficient showing increases activity. Statistical analysis was performed using 2-way ANOVA, with and without repeated measures for panels **C**–**G**, respectively, followed by Sidak’s post hoc test. Data are presented as mean ± SEM; significance set at *p* < 0.05. N = 19–23 mice per group. *p denotes *p* < 0.05, **p < 0.01, ****p < 0.0001

For the open field test, mice were run on two consecutive days. Conceptually, day one served the purpose of familiarizing the animals to the open field arena so that we can diminish the novelty of this arena upon subsequent exposure on day 2, which should presumably allow us to examine their basal ambulatory behavior (Fig. 5A, B). Implicit in this approach is the idea of habituation, which could be defined as a decrement in the amplitude of response due to repeated exposure, but not motor fatigue [45, 46]. Consequently, habituated animals should demonstrate decreased levels of spontaneous ambulatory activity when re-introduced into the open field arena.

Our open field data in females show that P2RY12-deficient mice exhibited hyperlocomotion (genotype, *F*_(1, 38)_ = 5.95, *p* = 0.02, Fig. 5C), with genotype-day interaction that is trending toward significance (interaction, *F*_(1, 38)_ = 3.79, *p* = 0.06, Fig. 5C). Pair-wise comparisons show that P2RY12-deficient female mice covered significant distance (*p* = 0.008, Fig. 5C) on day two when compared with their P2RY12-sufficient littermates. In males, however, there is no difference in the distance traveled between P2RY12-deficient mice and their littermate P2RY12-sufficient mice (genotype, *F*_(1, 40)_ = 1.27, *p* = 0.27, Fig. 5D), neither is there a main effect due to genotype-day interaction (interaction, *F*_(1, 40)_ = 1.34, *p* = 0.25, Fig. 5D). Pair-wise comparisons show no difference between P2RY12-deficient males and their P2RY12-sufficient. To further define the underlying mechanisms driving the hyperlocomotion, we observed a main effect in velocity due to genotype (genotype, *F*_(1, 38)_ = 5.74, *p* = 0.02, Additional file 2: Figure S2 A) and genotype-day interaction (interaction, *F* (_1, 38)_ = 4.32, *p* = 0.04, Additional file 2: Figure S2 A). Pair-wise comparisons show that P2RY12-deficient females have a higher (*p* = 0.008, Additional file 2: Figure S2 A) velocity when compared to their P2RY12-sufficient littermates on day two. In contrast, among males, our analysis showed neither genotype (genotype, *F*_(1, 40)_ = 2.09,* p* = 0.16 Additional file 2: Figure S2 B) nor genotype-day interaction (interaction, *F*_(1, 40)_ = 1.03, *p* = 0.32, Additional file 2: Figure S2 B) effect on male velocity. Also, pair-wise comparisons in males did not show a significant difference in velocity between P2RY12-deficient and P2RY12-sufficient mice, indicating a similar velocity regardless of genotype.

To further zoom in on their basal locomotor behavior on day two, the temporal progression of their locomotor behavior was determined by binning the outcome measures (distance traveled (Fig. 5E and F) and velocity (Additional file 2: Figure S2C and D) at 5 min intervals. According to our data, we find that P2RY12-deficient females exhibited an increase in total distance traveled (genotype, *F*_(1, 38)_ = 7.84, *p* = 0.008, Fig. 5E) with significant genotype-time interaction (interaction, *F*_(1, 190)_ = 2.59, *p* = 0.03, Fig. 5E). Our pair-wise comparisons across different time epochs indicated significant differences in distance traveled at the 25-min (*p* = 0.03, Fig. 5E) and 30-min (*p* = 0.008, Fig. 5E) marks. These findings suggest that P2RY12-deficient females may exhibit a deficit in intra-session habituation, as evidenced by their sustained activity levels in the open field arena without signs of desensitization over time.

Because we had previously observed effects of the estrus cycle on P2RY12 expression in wildtype female mice (Fig. 1E), we also examine whether there are differences in P2RY12-deficient female mice with regards to their estrus cycling that could explain the behavioral phenotypes. However, P2RY12-sufficient and P2RY12-deficient mice showed similar coefficient of variation— a measure of intrinsic variability within a group (Additional file 3: Figure S3).

In male mice, there were no significant differences in the distance traveled between P2RY12-deficient and P2RY12-sufficient counterparts (genotype, *F*_(1, 40)_ = 2.71, *p* = 0.10, Fig. 5F). Similarly, our analysis revealed no significant effect of genotype-time interaction on distance traveled (interaction, *F*_(5, 200)_ = 0.45, *p* = 0.81, Fig. 5F). Our results also show that P2RY12-deficient females displayed a main effect due to genotype (genotype, *F*_(1, 38)_ = 7.88, *p* = 0.008, Additional file 2: Figure S2C) and genotype-time interaction (interaction, *F* (5, 190) = 2.60, *p* = 0.03, Additional file 2: Figure S2C) on velocity. Pair-wise comparisons across different time epochs revealed that P2RY12-deficient females notably increased their velocity, especially at 25-min (*p* = 0.03, Additional file 2: Figure S2C) and 30-min (*p* = 0.008, Additional file 2: Figure S2C) marks. In contrast, no significant main effects due to genotype (genotype, *F*_(1, 40)_ = 3.69, *p* = 0.06, Additional file 2: Figure S2D) and genotype-time interaction (interaction, *F*_(5, 200)_ = 0.42, *p* = 0.83, Additional file 2: Figure S2D) were observed between male P2RY12-deficient mice and their P2RY12-suffcient littermates.

Lastly, to assess the sex-specific impact on basal locomotion (day 2), in light of the observed sex difference in P2RY12 expression levels (*p* = 0.0002, Fig. 1D), we conducted a sex-genotype interaction analysis. This analysis revealed no sex-specific effects on either the distance traveled (interaction, *F*_(1, 78)_ = 1.02,* p* = 0.31, Fig. 5G) or velocity (interaction, *F*_(1, 78)_ = 0.90, *p* = 0.34, Additional file 2: Figure S2E). However, we observed a significant main effect of genotype on both the distance traveled (genotype, *F*_(1, 78)_ = 9.17, *p* = 0.003, Fig. 5G) and velocity (genotype, *F*_(1, 78)_ = 10.67, *p* = 0.002, Additional file 2: Figure S2E). Pair-wise comparisons further indicated that P2RY12-deficient females traveled a greater distance (*p* = 0.03, Fig. 5G) and exhibited increased velocity (*p* = 0.02, Additional file 2: Figure S2E) compared to their P2RY12-sufficient littermates.

In examining basal locomotion further, we observed that both male and female P2RY12-deficient mice spent similar amounts of time in the center of the open field arena when compared to their P2RY12-sufficient littermates (Additional file 4: Figure S4A). Interestingly, while there was no significant difference in latency to reach the center among females regardless of their P2RY12 status, P2RY12-deficient males exhibited reduced latency to venture into the center than their P2RY12-sufficent littermates (Additional file 4: Figure S4B).

Collectively, our study shows that P2RY12-deficient females exhibited a significant increase in locomotor activity, as evidenced by their heightened basal locomotion on day 2 compared to P2RY12-sufficent littermates. This observation, set against a backdrop of intact gross motor coordination and balance (Additional file 5: Figure S5A–H), suggests a potential role for microglial P2RY12 in the regulation of locomotion. Importantly, our findings point to a sex-specific effect, where the phenomena of hyperlocomotion habituation deficit, observed in P2RY12-deficient females, does not appear in male counterparts. This distinction underscores the complexity of P2RY12’s involvement in locomotor behavior and highlights the need for further investigation into the sex-dependent mechanisms underpinning this regulation.

### P2RY12-deficient females exhibit anxiety-like response in the elevated plus maze (EPM)

**Fig. 6:**
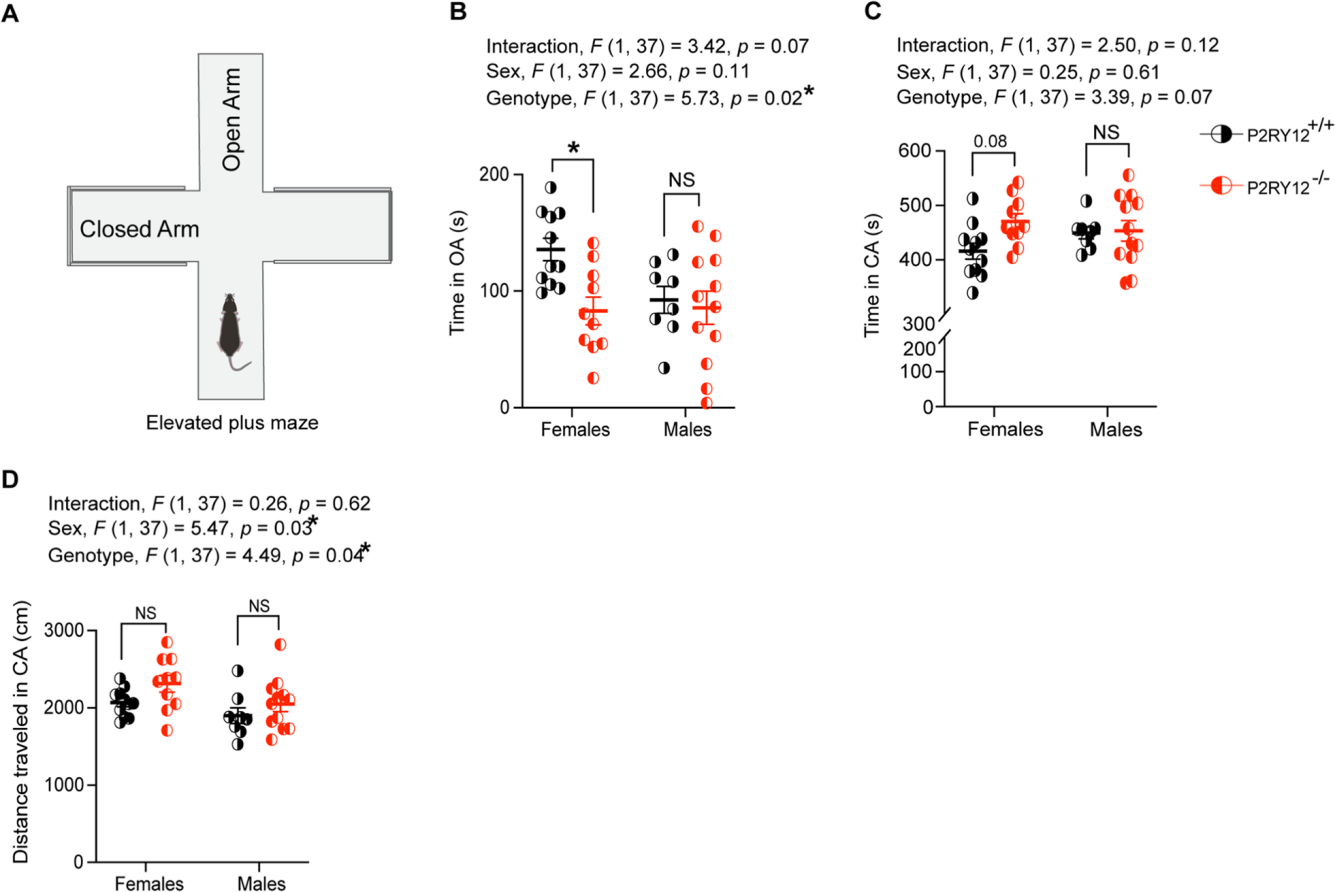
Anxiety-like behaviour in elevated plus maze reveals open arm avoidance by P2RY12-deficient female mice. **A** A sketch of the elevated plus maze (EPM) used to assess anxiety-like behaviour. **B** P2RY12-deficient females displayed a significance preference for avoiding the open arms. A trend towards a sex-genotype interaction was also observed. **C** While trends were noted in the effects of genotype and in post hoc analysis among females, no significance difference was found in the time spent in the closed arms of the EPM. **D** Analysis revealed main effects of genotype and sex on the distance traveled in the closed arm. Statistical analysis was performed using 2-way ANOVA followed by Tukey’s multiple comparisons test. Data are presented as mean ± SEM, with *p* < 0.05 indicating statistical significance. N = 8–12 mice per group. *p denotes *p* < 0.05. OA = open Arm, CA = closed Arm

Our data show that P2RY12 deficiency affects microglial morphology and dendritic spines in the hippocampus, with significant sex-genotype interactions (Figs. 3 and 4). This sex-specific cellular perturbations signifies a break down in microglial-neuronal interactions. Moreover, previous research has suggested that microglia and their interactions with neurons are important for various aspects of behavior, including anxiety-like behavior [10]. Therefore, it is reasonable to investigate whether deleting P2RY12 affects anxiety-like behavior in both male and female mice using the elevated plus maze task (Fig. 6A). With a separate cohort of mice that are littermates, our analysis identified a significant genotype effect (genotype, *F*_(1,37)_ = 5.73, *p* = 0.02, Fig. 6B), with P2RY12-deficient females spending less time in the open arms of the elevated plus maze (*p* = 0.03), indicative of anxiety-like behavior. This trend was not observed in P2RY12-deficient male mice compared to their wildtype littermates. Additionally, the interaction between sex and genotype in the time spent in the open arms approached significance (interaction,* F*_(1,37)_ = 3.42, *p* = 0.07, Fig. 6B). The analysis of time spent in the closed arm revealed a trend toward genotype effect (genotype, *F*_(1,37)_ = 3.39, *p* = 0.07, Fig. 6C). Specifically, P2RY12-deficient females exhibited a trend toward an increased duration in the closed arms of the maze (*p* = 0.08). Interestingly, significant main effects were observed for both genotype (genotype, *F*_(1, 37)_ = 4.49, *p* = 0.04, Fig. 6D) and sex (sex, *F*_(1, 37)_ = 5.47, *p* = 0.03, Fig. 6D) regarding the distance traveled in the closed arms, highlighting the influence of both genetic and sex differences within the maze. Collectively, our result suggests that in addition to locomotor hyperactivity, P2RY12-deficient females showcase anxiety-like behavior.

## Discussion

Microglia exhibit sexual dimorphism, as evidenced by several studies [56–58]. *P2ry12* is robustly expressed among microglia sensome genes in the basal condition [24]. *P2ry12* encodes the P2RY12 receptor, which was recently shown through ultrastructural analysis to mediate microglial-neuronal interactions both in the superficial and deep parts of the brain under basal condition [12]. Given these findings, we sought to ascertain the physiological significance of P2RY12-mediated microglial-neuronal interactions in a sex-specific context. In our study, we observed that adult female mice express greater levels of P2RY12 protein compared to their male counterparts, suggesting the possibility of hormonal influence, which we subsequently confirmed.

Given this differential expression in P2RY12 in adult microglia, we hypothesized that the loss of this gene might manifest in sex-specific cellular consequences and behavioral anomalies. Fascinatingly, our results revealed pronounced differences between P2RY12-sufficient and P2RY12-deficient mice. Firstly, we observed a sex-independent increase in microglial density with concomitant alteration in their spatial organization. Secondly, depending on the brain region, other cellular perturbations were either exclusive to females, or when present in both sexes, were markedly more pronounced in females. Specifically, we identified that: (I) microglial processes were more de-ramified in P2RY12-deficient females, and (II) neuronal dendritic spine density was reduced in P2RY12-defcient females. These anatomical perturbations in microglia and neurons suggest a breakdown in P2RY12-mediated microglial-neuronal interactions. Additionally, P2RY12-deficient females, but not males, display locomotor hyperactivity in the open field locomotor test and anxiety-like behavior in the elevator plus maze task. Overall, our findings suggest sex-specific roles for the P2RY12 that could be relevant for microglial-neuronal interactions.

### Impact of sex on P2RY12 expression in adult brain

Although the data points presented in Fig. 1C, D were obtained at different time points, the line graphs were constructed for descriptive purposes only, aiming to provide a visual representation of the trend over time. Here we show an initial steep rise in microglial P2RY12 expression in both male and female microglia from P7 to a peak at P21, as shown in Fig. 1D. This period coincides with significant developmental changes in the brain, including the rapid proliferation of neural cells and microglia [26]. Despite limited knowledge about P2RY12’s developmental role, recent findings from Cserép et al. [14] demonstrates that P2RY12 is important for the development and maintenance of a unique somatic purinergic interaction sites between microglia processes and the somas of developing neurons. This somatic junction appears to play critical roles in development, as P2RY12-deficient animals display layer-specific cortical dysgenesis. This suggests that the observed peak in P2RY12 expression during development may reflect its essential function in brain maturation.

The subsequent decrease in P2RY12 expression from adolescence to adulthood, alongside the observed sex-specific differences in adults, adds another layer of complexity to our understanding of P2RY12’s regulatory mechanisms. This trend parallels changes in microglial density observed in our unpublished data; yet it is important to note that our P2RY12 expression analysis was conducted on a per-cell basis, underscoring the nuanced regulation of this receptor throughout development. Moreover, the emergence of sex-specific P2RY12 expression patterns in adulthood, suggests that hormonal differences, particularly those linked to the maturation of ovarian function post-adolescence, may be involved in modulating P2RY12 expression. This hypothesis is corroborated by our observations of hormonal fluctuations during the estrus cycle significantly affecting P2RY12 expression in wildtype females (Fig. 1E).

Although the underlying mechanisms driving the peak in P2RY12 expression during the estrus and metestrus phases remain unclear, it is plausible to interpret this increase as a multifaceted adaptation. This adaptation likely responds to the complex interplay of hormonal, metabolic, and neuronal activity changes occurring during these periods. For example, the reproductive cycle involves significant hormonal fluctuations, which can affect neuronal activity and brain function. Increased P2RY12 expression during estrus and metestrus may represent an adaptive mechanism to maintain homeostasis and protect the brain from potential disturbances in neuronal activity associated with these hormonal changes. Specifically, it suggests that estrogen might upregulate P2RY12 expression as part of its extensive role in neuroprotection and maintaining brain health. This understanding of P2RY12’s regulation and function under normal physiological conditions raises intriguing questions about the consequences of its deficiency, particularly in the context of microglial-neuronal interactions.

### Impact of P2RY12 deficiency on microglial phenotype

Considering this sex difference in P2RY12 expression, we next explored how genetic disruption of this receptor could lead to distinct outcomes in males and females. Microglia, the primary resident immune cells in the brain parenchyma, possess a range of proteins enabling dynamic interaction with brain changes. The impact of P2RY12 deficiency on these cells may therefore illuminate further the receptor’s role in mediating the brains response to internal and external stimuli, potentially offering insights into sex-specific vulnerability in neuroimmune regulation. Because of their reactive nature as immune cells, there are checkpoint mechanisms in place to prevent their responses from going unchecked [17]. One mechanism involves how microglia interact with neurons. For example, a study by Neumann et al. (1996) found that microglia touching healthy neurons in organotypic slices did not become inflamed with IFN-γ treatment. Only microglia on the edges or near degenerating neurons became activated, thus suggesting the importance of cell–cell interaction as a key factor in microglial immune response. Conversely, recent findings by Cserép et al. [12] demonstrate that adult mice lacking P2RY12 have reduced interactions with neuronal elements in the resting state, indicating that P2RY12 plays a crucial role in facilitating microglial-neuronal interactions under normal conditions. This receptor likely allows microglia to monitor neuronal health or activity [3]. Here, in this study we report a sex-independent increase in microglial density and distribution across all examined brain regions in P2RY12-deficient mice (Fig. 2C–H). This occurred despite the variations in P2RY12 expression levels between male and female mice, suggesting that its role in regulating microglial population density operates independently of sex.

The findings highlight the intricate relationship between P2RY12 receptor expression and its regulatory role in microglial dynamics, posing a critical question: are changes in microglial density governed more by cell-intrinsic factors, like genetics, or by external influences, such as neural activity? The answer likely involves a synergy of both intrinsic and extrinsic factors that together dictate microglia behavior. Understanding this delicate balance is important, as it could unravel new dimensions of microglial-neuronal interactions and its implications for brain health.

Building on this exploration, we also observed that P2RY12 deficiency leads to significant morphological changes in microglia, such as de-ramification and shortened process lengths (Fig. 3C–H), changes that are especially marked in females. Our findings highlight a sex-specific effect of P2RY12 deficiency on microglial morphology, prompting an investigation into why females are more impacted than males. This difference may arise from sex-specific regulatory mechanisms of microglial morphology, possibly due to unique interactions between P2RY12 and pathways that differ by sex. Results from Villa et al. [58] support this notion, showing distinct gene expression profiles between male and female microglia, with genes related to anatomical structure and actin cytoskeleton organization notably enriched in females. Such differences suggest that male and female microglia possess inherent variations in the molecular frameworks that shape their morphology.

Surprisingly, we also find that P2RY12-deficient microglia in some regions—striatum and hippocampus—in males remain intact morphologically (Fig. 3G, H). Apparently, there are some regional differences in the expression of purinoceptors in the brain providing evidence of microglial regional heterogeneity. For example, there are a subset of microglia in the neurogenic subventricular zone (SVZ) that are incapable of eliciting ATP-inducible chemotaxis because they are deficient in purinoceptors [47, 60]. More pertinently, a recent work [35] looking at regional expressions of genes across different species, available at proteinatlas.org, shows that there are differences in the regional expression of P2RY12 that is maintained across species, including human, pig, and mouse. Therefore, it is possible that there could be other mechanisms in these regions in males besides P2RY12 that contribute to regulating microglial morphology. Essentially, the notion that male and female microglia may have some unique sex-specific mechanisms for regulating their morphology coupled with the differential regional expression of P2RY12 may explain why the male striatal and hippocampal P2RY12-deficient microglial morphology remain intact.

Our findings align with an emerging framework that recognizes sex-specific vulnerabilities in microglial morphology, similar to observations in various neurodegeneration models. Studies by Brunialti et al. [8] and Colombo et al. [11] demonstrate that, in the context of neurodegenerative stimuli, female microglia not only transition more rapidly to an affected morphology but are also more severely impacted than their male counterparts. This pattern mirrors the sex-specific response to P2RY12 deficiency we observed, suggesting that microglia may exhibit inherent sex-based differences in their vulnerability or response mechanisms to neurodegenerative challenges. Such insights underscore the importance of considering sex-specific factors in both the study of neurodegenerative diseases and the development of targeted strategies, highlighting the need for a nuanced approach to understanding these conditions.

### Impact of P2RY12 deficiency on neuronal phenotype

Given this complex background of microglial sex-specific responses, we turned our attention to the neuronal phenotype to further elucidate how P2RY12 deficiency affects neurons. Our analysis in the frontal cortex and dorsal striatum revealed significant main effects due to genotype and sex, respectively (Fig. 4C and E), indicating that the disruption extends beyond microglial changes to influence neuronal density. Further, we observed a significant sex-genotype interaction affecting dendritic spine density across all three regions examined (Fig. 4H–J). Notably, there is a reduction in striatal dendritic spines density in P2RY12 deficient females compared to their P2RY12-sufficient counterparts, a change not observed in males. This sex-specific structural alteration in the striatum coincides with observed microglial morphological differences in the same region, hinting at a tightly intertwined disruption in microglial-neuronal communication and structural integrity. The striatum is well known for its crucial role in motor control [20, 28], and sex-specific structural defects in the striatum could lead to differences in how males and females perform tasks related to locomotion. While previous studies [6, 51] have connected P2RY12 to experience-dependent synaptic plasticity, our findings broaden its significance to include basal synaptic regulation, showcasing a clear sex-dependent modulation. This discovery of P2RY12’s sex-specific modulation not only sheds light on critical aspects of microglial-neuronal interactions but also hints at there being variations that could be relevant for CNS disease susceptibility across sexes. Such molecular insights pave the way for examining how these cellular differences manifest in observable behaviors.

### Impact of P2RY12 deficiency on behavior

Transitioning from cellular mechanisms to behavioral outcomes, our data reveal a distinct sexual dimorphism, indicating clear differences between females and males (Figs. 5 and 6). Specifically, in the open field locomotor test, P2RY12-deficient female mice, unlike their male counterparts, exhibited locomotor hyperactivity (Fig. 5C–G). This hyperactivity can often be misinterpreted as impulsivity, but it is important to differentiate between these two behavioral outcomes. Impulsivity typically refers to a tendency to act on a whim, displaying behavior characterized by little consideration for the consequences. In the context of our study, impulsivity would have been indicated by a decreased latency to enter potentially threatening area such as the center of the open field arena. However, our data does not support this. The P2RY12-deficient females did not show a reduced latency to enter the open field’s center, suggesting that while they have a more pronounced locomotion, they do not necessarily act without caution. Both genotypes in females responded similarly to novelty-induced hyperlocomotion as seen in session one. In session two, however, we see a significant drop in the distance traveled in our P2RY12-sufficient mice relative to their P2RY12-deficient littermates. Seeing how the trajectory of their locomotion is divergent, one could infer that P2RY12-deficient mice are unable to decrease the amplitude of their response when re-exposed to the arena on session two—an indication of habituation deficit. When we binned their basal velocity over time (during session two), we see that the P2RY12-deficient females showed a time-specific increase in their velocity, a motor program under the control of the dorsal striatum [4, 20, 49]. The dorsal striatum, crucial for regulating locomotion [5, 28], may exhibit dysfunctional inhibitory pathways in P2RY12-deficient female mice, potentially underlying their hyperlocomotory behavior. This observation prompts an important question: why do only female P2RY12-deficent mice show locomotor hyperactivity, while males do not? Our findings suggest a sex-specific impact of P2RY12 deletion, notably with the male dorsal striatum appearing unaffected by the deletion. This difference could underlie the absence of altered locomotor behavior in male mice, as the intact inhibitory control within their dorsal striatum might protect against hyperactivity observed in females when re-exposed to the open field arena.

Our findings from the separate analysis using WT females in the first cohort showed that there was no significant effect of estrous cycle on the distance traveled in the open field. This observation is consistent with results from other studies that have used larger sample sizes and monitored estrous cycle and locomotor activity for more than 2 days [32, 38], further supporting the idea that the estrous cycle does not significantly influence locomotor behavior in the open field. Additionally, we found that P2RY12 deletion did not affect the cyclicality of estrous phases and Fischer’s exact test showed no significant difference between P2RY12^+/+^ and P2RY12^−/−^. But P2RY12^−/−^ displayed hyperlocomotion on day 2.

Additionally, we see that in the elevated plus maze, P2RY12-deficent females but not males showed anxiety-like behavior, and trends toward increased activity in the closed arm of the maze (Fig. 6B–D). This open arm avoidance in P2RY12-deficient females might be heightened due to their impaired habituation. That is, they might be more sensitive to the novel environment of the maze, leading to increased open arms avoidance, especially given that their increased level of activity in the closed arm of the maze. It is not uncommon for anxiety-like symptoms to co-occur with hyperactivity in neuropsychiatric disorders in humans and given the sex-specific effect of P2RY12 deletion across the brain regions examined with females being more dramatically impacted, this behavior seem to map onto the cellular deficits seen in these mice.

Although our elevated plus maze data contradict the result from Peng et al. [43], we have taken meticulous care in our methodology to ensure the accuracy of our findings. It is important to note that variations in experimental protocols are not uncommon and can significantly influence the outcomes of behavioral experiments. For instance, while Peng et al. [43] acclimated their mice for 30 min, we extended this acclimation period to 1 h, believing that a longer acclimation could impact the mice’s behavior. Additionally, our test duration was 10 min, in contrast to the 5-min duration used by Peng et al. [43]. These methodological differences, along with other subtle variations in experimental conditions such as lighting, age of mice, and environmental factors like housing conditions, diet, or even microbiome, can differ significantly between research facilities. Such variations are likely contributors to the differing results observed between our study and that of Peng et al. [43].

## Conclusion

Our study elucidates the complex role of P2RY12 in mediating sex-specific microglial and neuronal phenotypes and their subsequent behavioral manifestations. Through comprehensive analysis, we observed that P2RY12 deficiency leads to notable increases in microglial density and changes in cellular morphology, with these effects being particularly pronounced in females. These cellular alterations correlate with behavioral changes, as P2RY12-deficient female mice exhibited significant locomotor hyperactivity and anxiety-like behaviors, underscoring a critical sex-specific impact of P2RY12 in neuroimmune interactions. Importantly, our findings suggest that the P2RY12 receptor plays vital role in maintaining neuronal integrity and microglial function, with its deficiency disrupting the delicate balance of microglial-neuronal interactions. This disruption likely contributes to the observed behavioral phenotypes, highlighting the importance of considering sex as a significant factor in neurobiological research. By revealing the nuanced roles of P2RY12 in the brain, our study adds a crucial layer of understanding to the neuroimmune mechanisms underlying CNS diseases susceptibility.

### Limitations of the study

While the developmental roles of P2RY12 remains largely unexplored, recent work from Cserép et al. [14] shed light on its significance. The study reveals that P2RY12 is crucial for establishing and maintaining unique purinergic interaction sites between microglial process and the somas of developing neurons. These somatic junctions appear to play a pivotal in cortical layering. Notably, animals that lack P2RY12 exhibit layer-specific cortical dysgenesis, underscoring the potential involvement of P2RY12 in neurodevelopmental regulation. Because our study was performed exclusively in global P2RY12-deficient (and sufficient) mice, we cannot completely rule out the possibilities that our findings are a result of developmental abnormalities arising from the constitutive loss of P2RY12.

Additionally, we acknowledge the potential contributions from non-microglial cells expressing P2RY12, such as platelets. However, we consider this possibility unlikely. This is based on the selective expression of P2RY12 in microglia within the brain, which is the primary site of the cellular and behavioral phenotypes we examined. Furthermore, the study by Peng et al. [43] demonstrated consistent innate fear in their male-centered study using both constitutive and inducible deletion of P2RY12. These approaches not only highlight the ongoing significance of P2RY12 but also emphasize its specificity in the observed phenotype. Nevertheless, future work will have to directly test microglial-specific roles using conditional and inducible genetic and pharmacological loss-of-function approaches.

Lastly, we acknowledge that the observed changes in microglial morphology, neuronal spine density, and behavior may appear loosely connected, our objective was to provide a comprehensive overview of the potential implications of P2RY12 deficiency across sexes. Moreover, these observations are facets of the larger puzzle that highlights the multifaceted roles of microglial P2RY12 in neuroimmune interaction and brain function.

## Materials and methods

### Animals and estrous cycle monitoring

All the animals of both sexes used in this study were on a C57BL/6J genetic background. Littermate mice were generated by pairing male and female P2RY12^+/-^ mice generated from crossings with wildtype and P2RY12^−/−^ mice. In some experiments, mice were CX3CR1^−GFP/+^ to facilitate identification of microglia. All genotype combinations were co-housed by sex under controlled humidity, temperature, and a 12:12 h light–dark cycle, and with food and water access ad libitum.

In this study, the estrous cycle of animals used for behavioral assessments, specifically the open field and elevated plus maze tests, was documented using vaginal cytology approach as described by Byers et al. [9]. Additionally, we investigated the potential impact of ovarian hormone fluctuations on basal locomotion in two separate cohorts of mice. The first cohort consisted of pure wild-type (WT) mice, and the numbers of animals in each phase of the estrous cycle were as follows: proestrus:3; estrus: 7; metestrus: 4; and diestrus; 6. In total we used 20 WT females. Because we used CX3CR1^−GFP/+^ mice for our microglial analysis, we also conducted estrous cycle monitoring and open field test in these mice: CX3CR1^−GFP/+^: P2RY12^+/+^ and CX3CR1^−GFP/+^: P2RY12^−/−^ (P2RY12^+/+^ versus P2RY12^−/−^). The number of animals in each phase of the estrous cycle were as follows: P2RY12^+/+^ group: proestrus: 3; estrus: 4; metestrus: 2; and diestrus: 3; P2RY12^−/−^ group: proestrus: 4; estrus: 3, metestrus: 1; and diestrus: 5.

It is crucial to mention that the same cohort of mice underwent both the elevated plus maze and accelerated rotarod tests, which were spaced one week apart to mitigate any immediate any carryover effects. Given the rotarod test’s sensitivity to the animals’ training history, as underscored by McIlwain et al. (2001), we prioritized it as the initial assessment in our series of behavioral tests.

### Ethics statement

All animal handling and procedures were performed in strict adherence to the established and approved guidelines and regulations of the Institutional Animal Care and Use Committee at the University of Virginia. Young (P7–P21) mice were sacrificed by cervical dislocation whereas adult (12–18 weeks old) mice were euthanized with CO_2_ for the following experiments. Essentially, all efforts were expended to reduce animal suffering.

### Flow cytometry

Mice were anesthetized with isoflurane and transcardially perfused with ~ 20 mL ice-cold 1X PBS. The brain was immediately dissected and finely minced with a scalpel to ~ 1 mm pieces and suspended in pre-warmed dissociation media (DMEM (Gibco, NY, US) + 10% FBS DMEM (Gibco, NY, US) + 1 mg/mL Collagenase VIII (Sigma-Aldrich, St Louis, MO, USA) + 0.5 mg/mL DNase I (Roche, Germany) for 1 h at 37 °C with constant agitation. After 30 min of incubation, the suspension was triturated gently with a 5 mL serological pipette. Subsequently, the suspension was centrifuged for 5 min at 1500x*g* at 4 °C, the supernatant was carefully decanted, and the pellet was resuspended in 10 mL of 37% isotonic Percoll (GE Healthcare) solution diluted with DMEM. The suspension was centrifuged for 10 min at 1000x*g* at 4 °C. The top floating myelin layer and supernatant were aspirated. The cells were washed with 10 mL of FACS buffer, centrifuged for 5 min at 1500xg, and the cell pellet was resuspended in 1 mL FACS buffer. The cell number was counted using an automated cell counter (C100, RWD, China). One hundred μL of resuspended cells were placed in a v-bottom 96-well plate (NEST, China) and spun down for 5 min at 1500xg. The supernatant was carefully decanted. Unspecific binding sites were blocked with 50 μL Fc block (1:1000, CD16/32, Clone 93, eBioscience) for 10 min at room temperature. Cells were then incubated in primary antibodies at a concentration of 1:200 and fixable viability dye eFluor 506 (eBioscience) at a concentration of 1:800 for 30 min at 4 °C. Mononuclear cells were labeled with ef780 mouse anti-CD11b antibody (eBioscience, clone: M1/70), PECy7 anti-mouse CD45 antibody (eBioscience, clone: 30-F11), and PE mouse anti-P2RY12 antibody (BioLegend, Clone: S16007D) for 30 min at 4 °C. While the CX3CR1^−GFP/+^ mouse model is a robust tool for identifying microglia, we wanted to ensure the highest level of accuracy in our cell population. The additional immunostainings served as a validation step, ensuring that the GFP-positive cells we were analyzing were indeed microglia. After staining, cells were washed twice and fixed overnight in 1% PFA at 4 °C. After overnight fixing, cells were washed twice with FACS buffer and transferred into a 5 mL Polystyrene round-bottom tube with cell-strainer cap tubes (Falcon). Post-staining fixation ensures the stability of the cells and allowing them to sit overnight ensures that they reach their final FSC, SSC, and fluorescence levels. The samples were then analyzed on a Gallios flow cytometer (Beckman-Coulter) by gating on 100,000 live events. Flow cytometry data were analyzed using FlowJo (version 10.8.1). Live mononuclear cells were gated based on fixable Viability Dye (eFlour 506)/Time, then gated based on FS-H/FS-A to identify single cells. GFP^+^ cells were gated based on FS-A/GFP; microglia were gated based on CD45/CD11b. P2RY12 expression was determined by geometric mean from microglial population determined by CD45^inter^CD11b^+^. It is worth mentioning that this methodology was repeated for each time point for the P2RY12 profiling in male and female across ages.

### Microglial density and morphology

Microglial population density and morphology were assessed in young adult mice (12 weeks old). For detection of microglia cells, we used mice that are either heterozygous for GFP expression driven by fractalkine receptor CX3CR1 as control (P2RY12^+/+^:CX3R1^−GFP±^) or in combination with mice that are deficient in P2RY12 as experimental group (P2RY12^−/−^:CX3CR1^−GFP±^). These mice were euthanized using CO_2_ and fixed by transcardial perfusion with 4% (wt./vol) paraformaldehyde (PFA); their brains were removed and post-fixed overnight in 4% PFA at 4 °C. Coronal section (40 μm) of the brains were cut using a vibratome (Leica VT1000S). Tissue sections were mounted on microscope slides and then cover-slipped with DAPI Fluoromount-G (Southern Biotech) mounting medium. Stack images (35–38 μm) with a z-step size of 1 μm were acquired using 40 × oil immersion objective (NA 1.4) on the SP8 Leica confocal microscope. Four to five slices per animal were sampled for analysis. Cell counting and nearest neighbor analyses were carried out using the “Spot” module in Imaris (version 9.7.0, Bitplane, Oxford Instruments). Three dimensional Sholl analysis with a sphere of resolution of 5 μm was performed according to steps previously outlined by Althammer et al. [1]. Briefly, surface rendering was performed using the following custom settings: Surface details: 0.80 μm; Thresholding: background subtraction (local contrast); Filter type: volume (400 μm^3^). Microglia cells that were incomplete, fused, or located within the 5 μm of the XY border were excluded from the analysis. After applying these exclusion criteria, we generated a new channel containing only the intact cells using the “Mask all” function in Imaris. For filament reconstruction, we applied the automatic algorithm. A filament consists of two elements, vertices (drawn as nodes or dots) and edges (lines connecting the vertices). For quantification, we followed the approach previously reported by Kyrargyri et al. (2020). Following the application of the abovementioned exclusion criteria, 6–12 cells per brain region were selected from 2 to 3 mice per group and analyzed. In total, 18–36 cells per group were used for the statistical analysis.

### NeuN immunofluorescent staining

Mice were first perfused with 1X phosphate buffered saline (pH = 7.4; Life Technologies Corporation, NY, USA) and then by 4% (wt./vol) paraformaldehyde (PFA) (Product #: 158127; Sigma-Aldrich, Germany) in phosphate buffer (PB), after which they were decapitated, and their brains harvested and post-fixed overnight in 4% (wt./vol) PFA. Then the brains were washed in PBS and stored at 4 °C. Forty μm thick free-floating sections were obtained using a vibratome (Leica VT1000S). Four sections per mouse were incubated in blocking buffer consisting of 10% normal donkey serum (Product #: 017-000-121; Jackson ImmunoResearch Laboratories, Inc. USA) and 0.3% triton X-100 solution (Product #: 93443; Sigma-Aldrich, Germany) in Tris-buffered saline (TBS) (Product #: BP24721; Thermo Fisher Scientific, MA, USA) for 1 h at room temperature. Anti-NeuN (Rat anti-NeuN, Product #: 279297, abcam, United Kingdom) primary antibody was prepared in blocking buffer at 1:500 dilution, and sections were incubated with primary antibodies overnight at 4 °C. Secondary antibody, donkey anti-rat Alexa Fluor 488, (Product #: A-21208, Thermo Fisher Scientific, USA) was also prepared in blocking buffer at a 1:500 dilution. Sections were then incubated in secondary antibody for 1 h. Cell nuclei were stained by incubating sections in HOECHST 33342 (Product #: R37605, Thermo Fisher Scientific, USA) for 30 min. Images were acquired using STELLARIS 5 confocal microscope (Leica Microsystems, USA) with a 20 × objective. Cell (NeuN) counting was performed using Imaris software (10.0.1).

### P2RY12 immunofluorescent staining

Mice were perfused with 1X phosphate buffered saline (pH = 7.4; Life Technologies Corporation, NY, USA) followed by 4% (wt./vol) PFA (Product #: 158127; Sigma-Aldrich, Germany) in phosphate buffer (PB). After perfusion, the mice were decapitated, their brains harvested and post-fixed overnight in 4% (wt./vol) PFA. Forty μm free-floating sections were obtained using a vibratome (Leica VT1000S). Four sections per mouse were incubated in blocking buffer consisting of 10% normal donkey serum (Product #: 017-000-121; Jackson ImmunoResearch Laboratories, Inc; USA) and 0.3% Triton X-100 solution (Product #: 93443; Sigma-Aldrich, Germany) in Tris-buffered saline (Product #: BP24721; Thermo Fisher Scientific, MA, USA) for 1 h. After blocking, sections were washed three times in TBS, 5 min each. The anti-P2Y12 primary antibody (Rabbit, pAb, Product #: AS55043A, AnaSpec, USA) was prepared in blocking buffer at a 1:300 dilution and incubated with the sections overnight at 4 °C. Following primary antibody incubation, sections were washed three times in TBS, 5 min each. The secondary antibody, donkey anti-rabbit Alexa Fluor 647 (Product #: A-31573, ThermoFisher Scientific, USA), was prepared at a 1:500 dilution in blocking buffer. Sections were then incubated in secondary antibody for 1 h at room temperature, followed by three washes in TBS, 5 min each. All sections were transferred to a fresh 1X TBS and incubated in HOECHST 33342 (Product #: R37605, Thermo Fisher Scientific, USA) for 30 min after which they were mounted on glass slides with ProLong Gold antifade mountant (Invitrogen, P36930) and coverslipped. Images were acquired using STELLARIS 5 confocal microscope (Leica Microsystems, USA) with a 40 × objective. Fluorescent intensity analysis performed using Fiji software.

### Golgi staining technique

Whole brain was harvested from adult mice (P90) and stained using the FD Rapid GolgiStain kit (FD Neuro Technologies, Inc. MD, USA). The brains were rinsed with deionized water and then properly immersed in the impregnation solution, which consist of equal volumes of solution A and B, for 2 weeks in the dark. The brains were then transferred into solution C and kept at room temperature in the dark for another week during which the solution was replaced twice. For vibratome sectioning, each impregnated brain was placed in a disposable base mold (Product #: 41743, fisherscientific, USA) and embedded in 4% (wt./vol) agarose/TAE. Sectioning was carried out using a vibratome (Leica VT1000S) with the buffer tray filled with solution C. One hundred μm thick coronal sections were cut and transferred to gelatin-coated microscope slides on small drops of solution C. Sections were allowed to dry in the dark at room temperature for 24 h, after which they were then stained according to the outlined procedure in the FD Rapid GolgiStain manual. Sections were also counterstained with cresyl violet to determine the locations of impregnated neurons and anatomical landmark visualization. Several sections were obtained from each mouse using 100x (plus oil immersion) objective on a Keyence microscope (BZ-X800, Osaka, Japan). A minimum of 60 μm thick z-stack images were taken from different regions of the brain of each mouse. We collected data from the cortex, hippocampal CA1, and the dorsal striatum of every mouse, considering both sex (male and female) and genotype (P2RY12-sufficent and P2Ry12-deficient) distinctions. Specifically, for each mouse in each group, we analyzed 15–20 secondary and tertiary dendrites (at least 50 μm away from the soma) per brain region using Fiji software (http://imagej.net). While the phases of the estrous cycle were not documented during this experiment, we acknowledge that this could potentially influence our result.

### Behavioral assessment

#### Open field locomotory test

The open field locomotory test was performed over the course of two days, with day 1 serving to habituate mice to the testing apparatus, which should presumably dampen novelty-induced hyperlocomotion upon subsequent re-exposure on day 2. This would then allow us to examine their innate basal locomotory behavior in the open field arena. Each day comprises two sessions with each session starting with an alternate sex. Our primary intention behind this scheme was to ensure both male and female mice have an even distribution of experience in the arena across the two days. With this approach, we aimed to control for any potential biases that might arise from one sex consistently being tested at a particular time of the day. Briefly the open field locomotory test was carried out in a custom-built arena [35 (L) × 35 (B) × 21 (H) cm] made with white plastic material. Only cages containing animals belonging to one sex category were moved into the dedicated behavior testing room, which was illuminated at an intensity that is < 150 Lux. The room temperature (71.8 ± 1.5 °F) and humidity (27.35 ± 0.5 °F) remained relatively constant throughout the testing period. The animals were allowed to acclimate to the testing room for 1 h before starting each session. Each run consists of 8 open field arenas and 1 mouse per arena. Each mouse was placed at the periphery of the arena and allowed to freely explore the homogenous space; their activities over the course of 30 min were monitored by 2 overhead cameras and simultaneously tracked using EthoVision XT (Noldus, Wagenigen, Netherlands), with the experimental blind to the genotype and behind a curtain barrier. After each experimental run, the arenas were thoroughly wiped clean with 70% ethanol to remove any residual fecal matter or lingering olfactory cues and allowed to air dry before running another test on the next set of 8 mice.

#### Accelerated rotarod test

The accelerated rotarod test was carried out over the course of three consecutive days. Each day comprises two sessions with each session starting with an alternate sex. The order in which the sex and the mice were exposed to the rotarod was determined by simple random sampling using the online GraphPad software. Only cages containing animals belonging to one sex category were moved into the dedicated behavior testing room, which was illuminated at an intensity that is < 150 Lux. The room temperature (71.5 ± 0.4°F) and humidity (34.63 ± 13 RH) remained relatively constant throughout the testing period. They were allowed to acclimate to the room for one hour before the test. Each mouse was placed on the accelerated rotarod (Med Associates, Inc, Vermont, USA) programmed to run from 4 to 40 rev/min for 5 min during which their latency to fall and speed were recorded. At the end of each run, the suspended cylindrical rod was thoroughly wiped clean with 70% ethanol to remove any residual fecal matter or lingering olfactory cues and allowed to air dry before running another test on the next set of 5 mice. The experimenter was blind to the genotype of the mice.

#### Elevator plus maze (EPM)

EPM was performed in a custom-made light grey (for the floor) and black (walls) plastic materials: the arm length is 33 cm, lane width is 5.8 cm, closed arm wall height is 20 cm, and the open arms wall height is 0.5 cm. The whole maze has an elevation height of 68 cm from the ground. The order in which the sex and the mice were exposed to the EPM was determined by simple random sampling using the online GraphPad software. They were allowed to acclimate in the room with 68.1 °F ambient temperature, 24.9 RH relative humidity, and < 150 Lux illumination for 1 h. Each mouse was placed in the hub of the EPM with the face toward the open arm. They were allowed to explore the maze for 10 min during which their activities were monitored with an overhead camera and simultaneously tracked using EthoVision XT (Noldus, Wagenigen, Netherlands). The experimenter was blind to the genotype and stayed behind a curtain barrier.

### Statistical analysis

All data are presented as mean ± SEM. Statistical analyses were performed using GraphPad Prism (v9.5.1 for Mac, GraphPad Software, Lajolla California, USA) or R (v4.2.1). Two independent group comparisons were carried out using two-tailed student’s t test and more than two groups by either 2-Way ANOVA or 2-Way repeated measures (RM) ANOVA, and where suitable with post-hoc analysis by either Tukey’s or Sidak’s pair-wise comparisons. P < 0.05 was taken as statistically significant.

### Supplementary Information

**Additional file 1. Flow experimental design and fluorescent intensity of cortical P2RY12 expression.** (**A**) Overview of the flow cytometry experimental design used to assess P2RY12 expression. (**B**) Gating strategy employed to differentiate P2RY12 expression levels between male and female mice. (**C**) Representative cortical images depicting P2RY12 expression in adult male and female mice. (**D**) Quantitative analysis of P2RY12 expression reveals no significant differences between sexes in the cortex, as determined by Student’s t-test. Data are presented as mean ± SEM for N = 4 mice per group. Scale bar: 10μm. FS-H (forward scatter height) and FS-A (forward scatter area) are included to detail the flow cytometry analysis parameters.

**Additional file 2. P2RY12-deficient females display time-specific increase in velocity.** (**A**) P2RY12-deficient females exhibit impaired velocity reduction across two days. (**B**) P2RY12-deficient males maintain consistent locomotor velocity over the same period. (**C**) A significant increase in basal velocity is observed in P2RY12-defienct females. (**D**) P2RY12-deficient males demonstrate basal velocities comparable to P2RY12-sufficient littermates. (**E**) A genotype-dependent effect is evident, with P2RY12-deficient females (but not males) showing increased basal velocity on day 2. Statistical analysis was performed using repeated measures 2-Way ANOVA for Panels A and B, and ordinary 2-Way ANOVA for Panel C to E, both followed by Šidák’s multiple comparisons test. Data are presented as mean ± SEM, with *p* <0.05 indicating statistical significance. N = 19–23 mice per group. Significance levels are denoted as *p <0.05, **p <0.01, and ****p <0.0001.

**Additional file 3. P2RY12-deficient females show increased basal (day 2) locomotion.** Cells in the vagina smear consist of (**A**) Nucleated epithelial cells, (**B**) Leukocytes, and (**C**) Squamous epithelial cells. Panel D–G indicates mice in (**D**) Proestrus, (**E**) Estrus, (**F**) Metestrus, and (**G**) Diestrus. (**H**) Shows the cycling state distribution in both P2RY12 WT and P2RY12 KO mice before the open field locomotor test. (**I**) Open field arena. (**J**) P2RY12-deficient females significantly covered more distance when compared to their P2RY12-sufficient counterpart. (**K**) Shows no difference in coefficient of variation. Panel H data is presented as #mice per estrous cycle phase and data analyzed by Fisher’s exact test. Panel J data are presented as mean ± SEM and analyzed using Student’s t test. Panel K is presented as percent of coefficient of variation. **p denotes *p* <0.01. N = 12/13 per group.

**Additional file 4.** Reduced latency to center in P2RY12-deficient male mice on day 2 in the open field test. (**A**) P2RY12-sufficient and –deficient mice showed no difference in time spent in the open field center. (**B**) A significant effect of sex and genotype was observed; P2RY12-deficient males, but not females, entered the center more quickly. Statistical analysis used 2-Way ANOVA with Tukey’s test, indicating significance at *p* < 0.05. Data are mean ± SEM, N = 19–23 per group. *p < 0.05, **p < 0.01.

**Additional file 5. No gross motor balance and coordination deficit in P2RY12 deficient mice following accelerated rotarod testing.** (**A**) Set up of accelerated rotarod test. (**B**) Diagram illustrating the experimental flow for conducting the accelerated rotarod test. (**C**) Comparable body weights in female mice across groups. (**D**–**E**) Latency to fall and rotational speed show no significant differences between P2RY12-sufficient and –deficient female mice over a 3-day period. (**F**) Male mice exhibit similar body weights regardless of P2RY12 status. (**G**–**H**) Shows no difference in latency to fall and rotational speed over the course of 3 days between P2RY12-sufficient and -deficient males. Student’s t test was applied for comparing body weights (**C**, **F**), while repeated measures 2-Way ANOVA, followed by Šidák’s multiple comparisons test (D, E, G, and H). N = 12–15 mice per group.

## Data Availability

All datasets used and/or analyzed in this present study are available from the corresponding author upon request.
