# Supplementary figures and images for: A P2RY12 deficiency results in sex-specific cellular perturbations and sexually dimorphic behavioral anomalies
Source: J Neuroinflammation. 2024 Apr 15;21:95. doi: 10.1186/s12974-024-03079-7 (PMC11017545; doi:10.1186/s12974-024-03079-7)

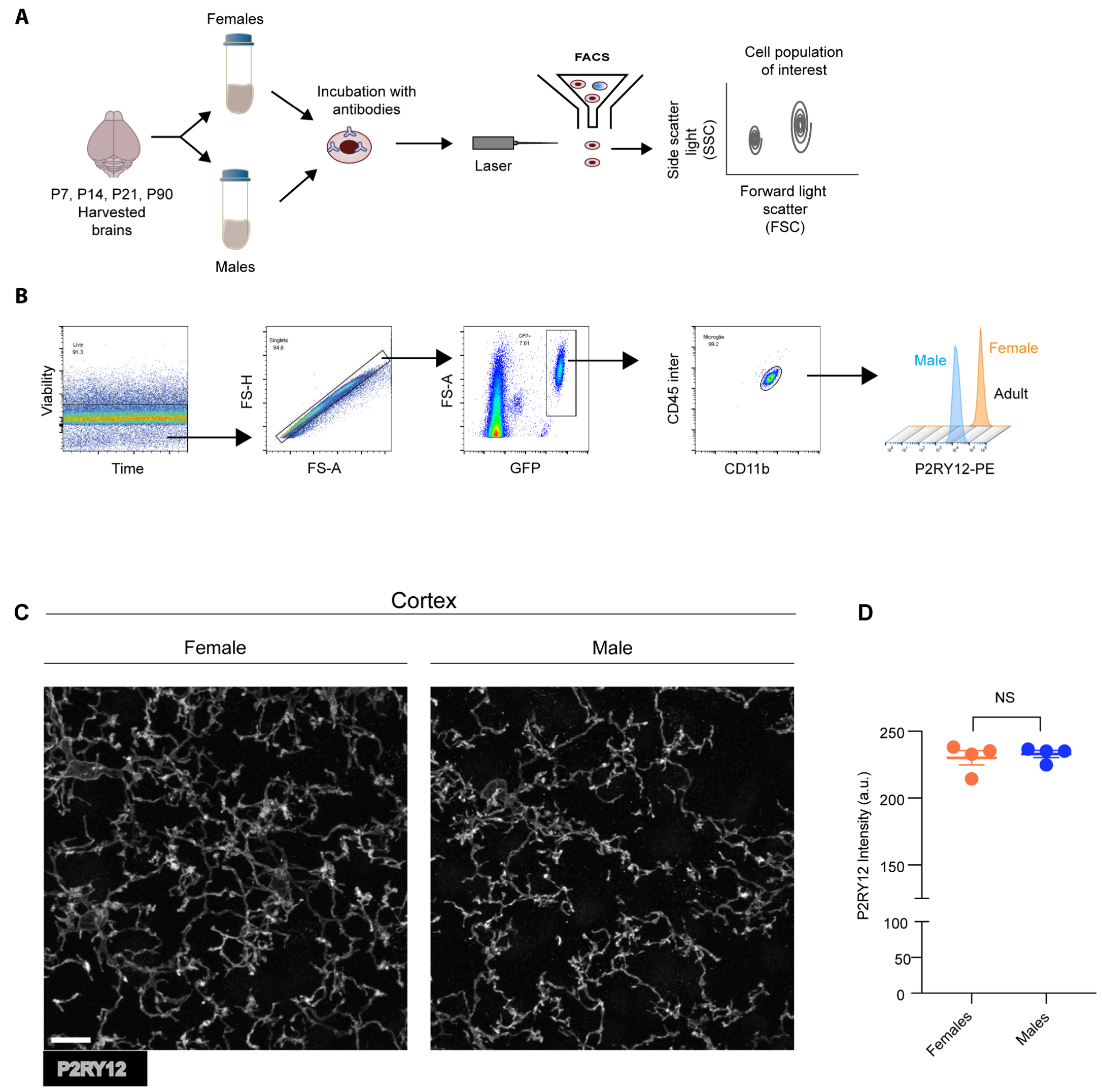

Supplement: Supplementary file 1 — Additional file 1. Flow experimental design and fluorescent intensity of cortical P2RY12 expression. (A) Overview of the flow cytometry experimental design used to assess P2RY12 expression. (B) Gating strategy employed to differentiate P2RY12 expression levels between male and female mice. (C) Representative cortical images depicting P2RY12 expression in adult male and female mice. (D) Quantitative analysis of P2RY12 expression reveals no significant differences between sexes in the cortex, as determined by Student’s t-test. Data are presented as mean ± SEM for N = 4 mice per group. Scale bar: 10μm. FS-H (forward scatter height) and FS-A (forward scatter area) are included to detail the flow cytometry analysis parameters. [file 12974_2024_3079_MOESM1_ESM.tiff]

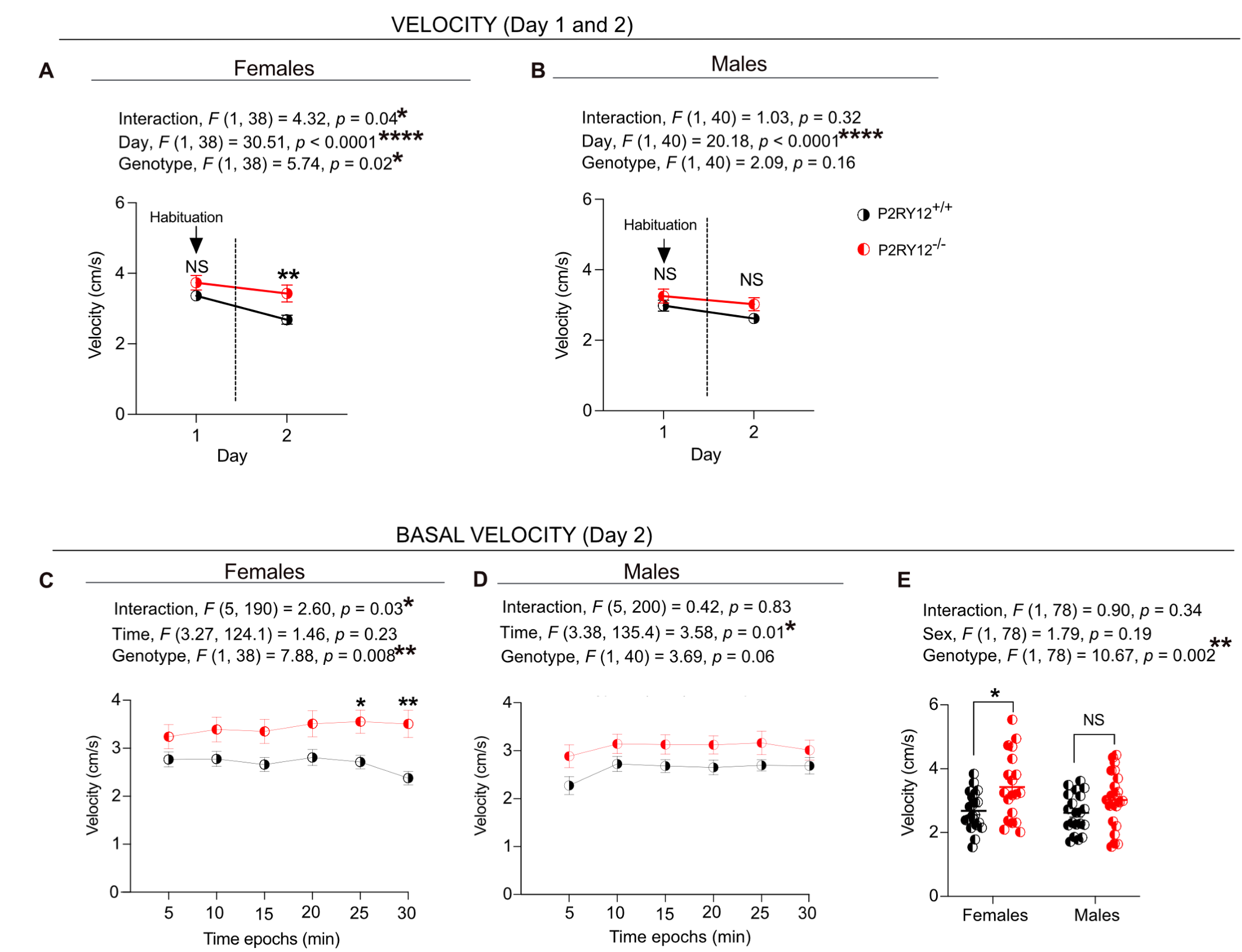

Supplement: Supplementary file 2 — Additional file 2. P2RY12-deficient females display time-specific increase in velocity. (A) P2RY12-deficient females exhibit impaired velocity reduction across two days. (B) P2RY12-deficient males maintain consistent locomotor velocity over the same period. (C) A significant increase in basal velocity is observed in P2RY12-defienct females. (D) P2RY12-deficient males demonstrate basal velocities comparable to P2RY12-sufficient littermates. (E) A genotype-dependent effect is evident, with P2RY12-deficient females (but not males) showing increased basal velocity on day 2. Statistical analysis was performed using repeated measures 2-Way ANOVA for Panels A and B, and ordinary 2-Way ANOVA for Panel C to E, both followed by Šidák’s multiple comparisons test. Data are presented as mean ± SEM, with p <0.05 indicating statistical significance. N = 19–23 mice per group. Significance levels are denoted as *p <0.05, **p <0.01, and ****p <0.0001. [file 12974_2024_3079_MOESM2_ESM.tiff]

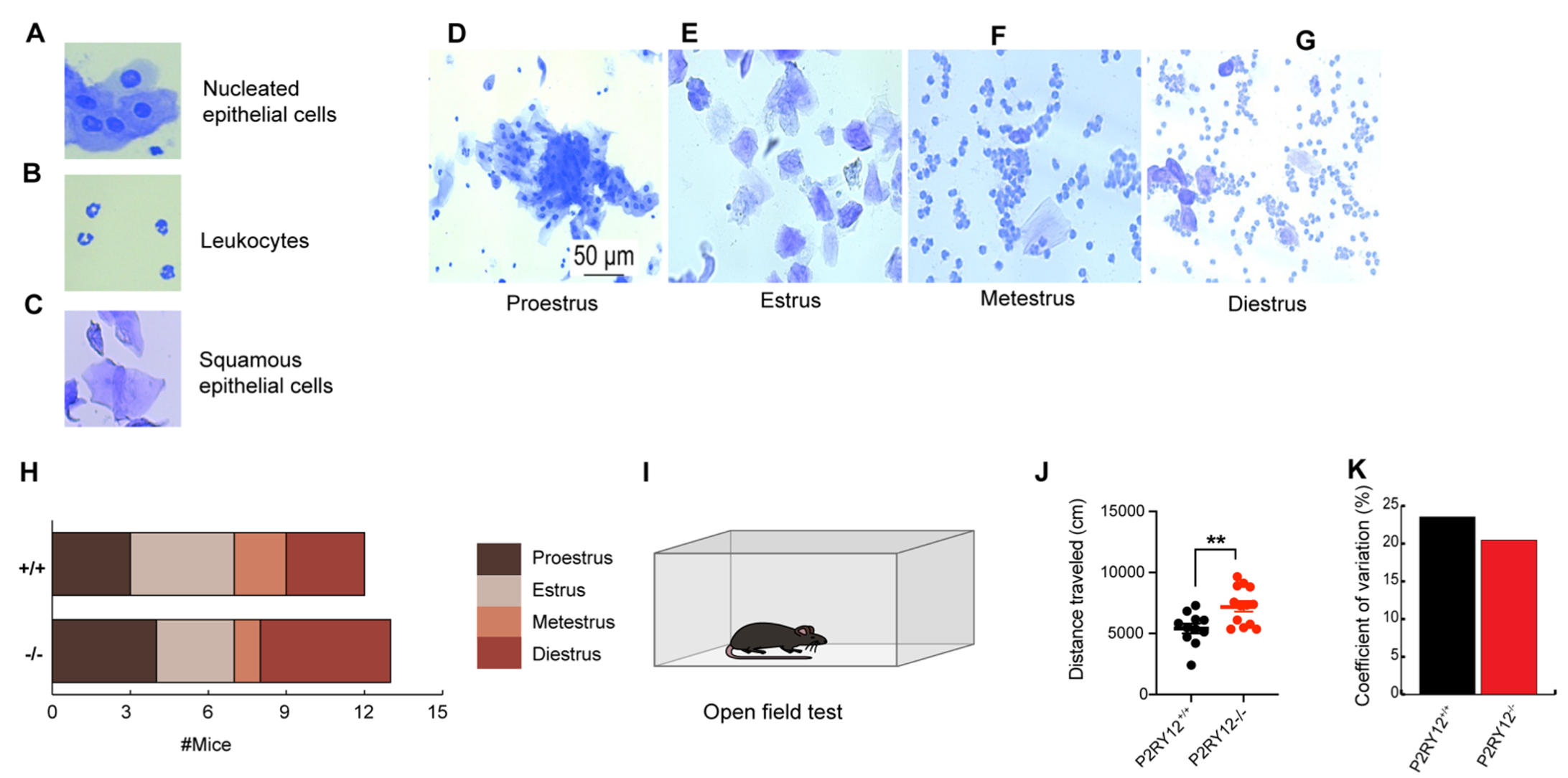

Supplement: Supplementary file 3 — Additional file 3. P2RY12-deficient females show increased basal (day 2) locomotion. Cells in the vagina smear consist of (A) Nucleated epithelial cells, (B) Leukocytes, and (C) Squamous epithelial cells. Panel D–G indicates mice in (D) Proestrus, (E) Estrus, (F) Metestrus, and (G) Diestrus. (H) Shows the cycling state distribution in both P2RY12 WT and P2RY12 KO mice before the open field locomotor test. (I) Open field arena. (J) P2RY12-deficient females significantly covered more distance when compared to their P2RY12-sufficient counterpart. (K) Shows no difference in coefficient of variation. Panel H data is presented as #mice per estrous cycle phase and data analyzed by Fisher’s exact test. Panel J data are presented as mean ± SEM and analyzed using Student’s t test. Panel K is presented as percent of coefficient of variation. **p denotes p <0.01. N = 12/13 per group. [file 12974_2024_3079_MOESM3_ESM.tiff]

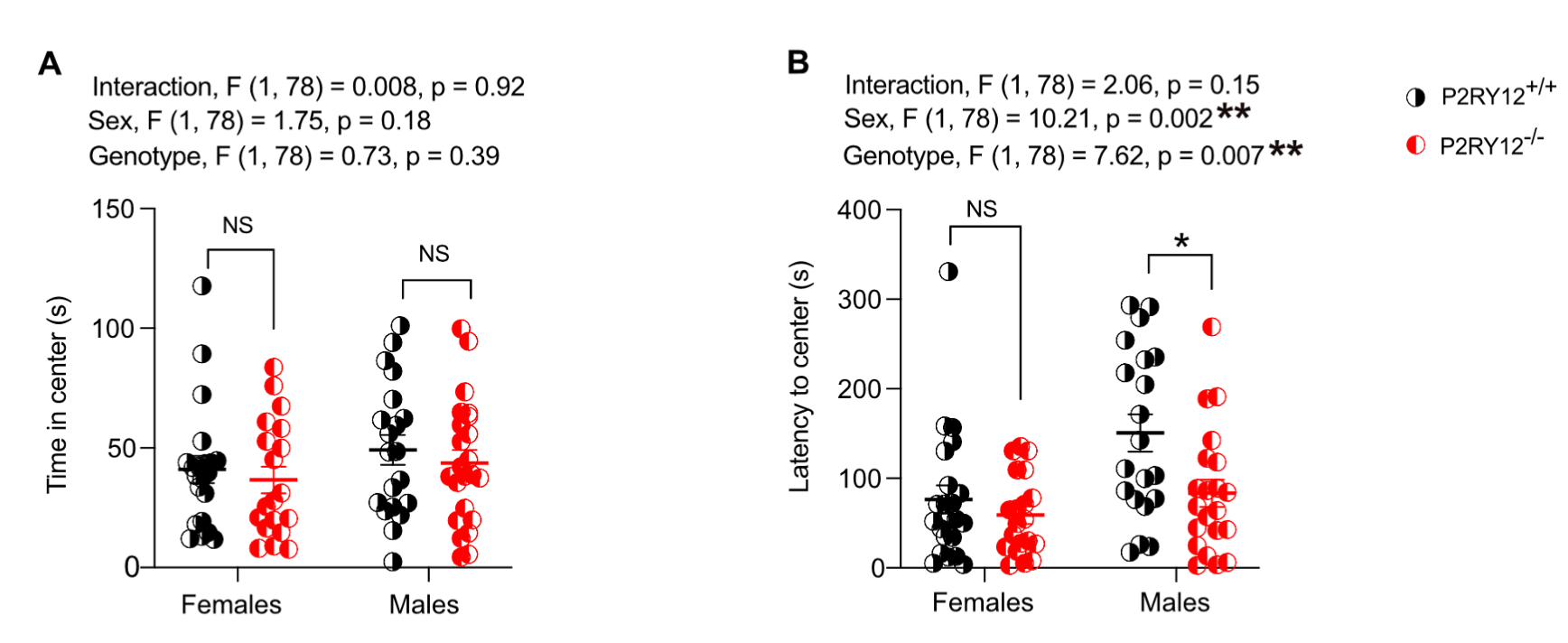

Supplement: Supplementary file 4 — Additional file 4. Reduced latency to center in P2RY12-deficient male mice on day 2 in the open field test. (A) P2RY12-sufficient and –deficient mice showed no difference in time spent in the open field center. (B) A significant effect of sex and genotype was observed; P2RY12-deficient males, but not females, entered the center more quickly. Statistical analysis used 2-Way ANOVA with Tukey’s test, indicating significance at p < 0.05. Data are mean ± SEM, N = 19–23 per group. *p < 0.05, **p < 0.01. [file 12974_2024_3079_MOESM4_ESM.tiff]

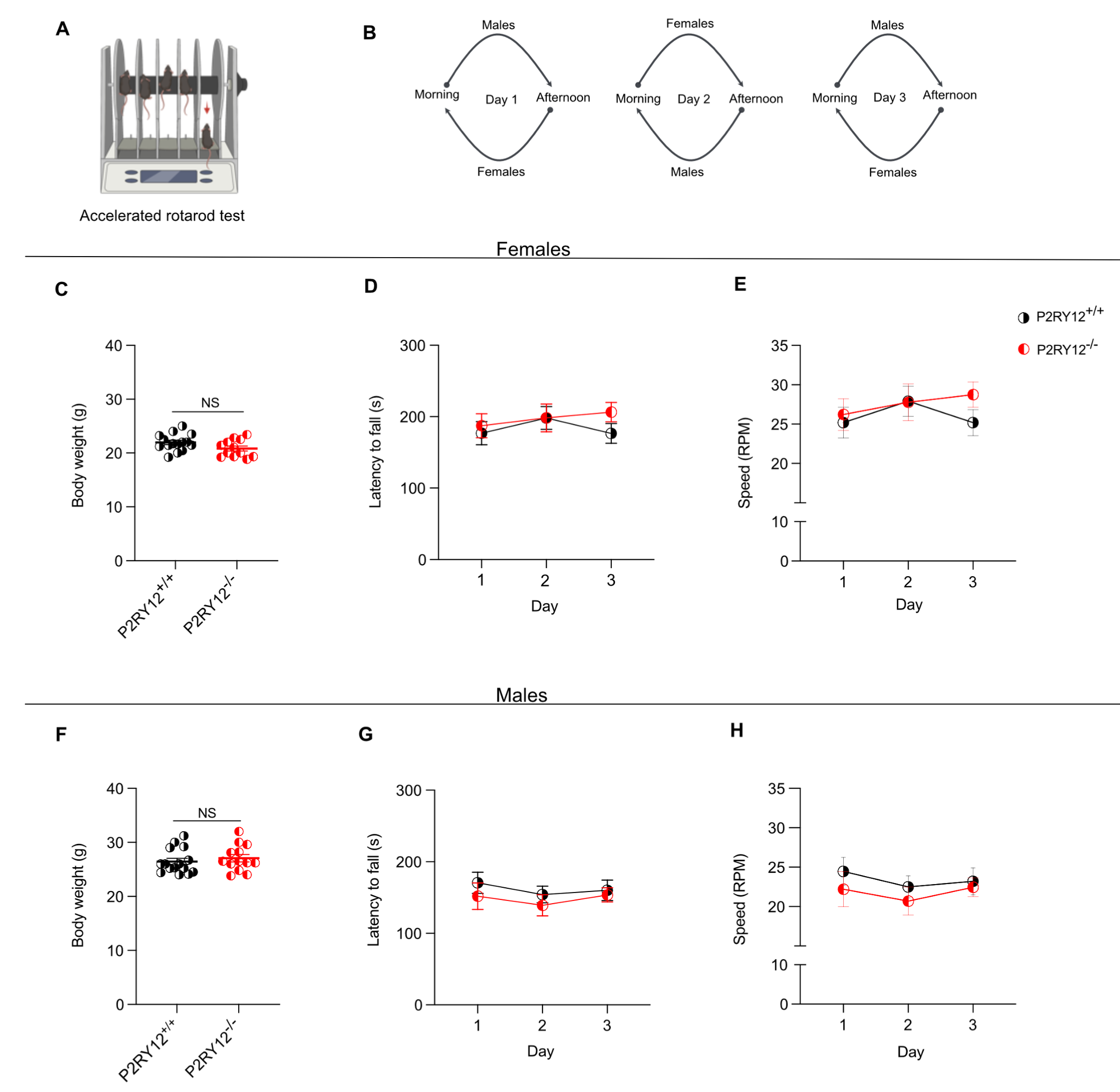

Supplement: Supplementary file 5 — Additional file 5. No gross motor balance and coordination deficit in P2RY12 deficient mice following accelerated rotarod testing. (A) Set up of accelerated rotarod test. (B) Diagram illustrating the experimental flow for conducting the accelerated rotarod test. (C) Comparable body weights in female mice across groups. (D–E) Latency to fall and rotational speed show no significant differences between P2RY12-sufficient and –deficient female mice over a 3-day period. (F) Male mice exhibit similar body weights regardless of P2RY12 status. (G–H) Shows no difference in latency to fall and rotational speed over the course of 3 days between P2RY12-sufficient and -deficient males. Student’s t test was applied for comparing body weights (C, F), while repeated measures 2-Way ANOVA, followed by Šidák’s multiple comparisons test (D, E, G, and H). N = 12–15 mice per group. [file 12974_2024_3079_MOESM5_ESM.tiff]
